# Toward Physics-Based Solubility Computation for Pharmaceuticals to Rival Informatics

**DOI:** 10.1021/acs.jctc.1c00130

**Published:** 2021-05-14

**Authors:** Daniel J. Fowles, David S. Palmer, Rui Guo, Sarah L. Price, John B. O. Mitchell

**Affiliations:** † Department of Pure and Applied Chemistry, University of Strathclyde, Thomas Graham Building, 295 Cathedral Street, Glasgow, Scotland G1 1XL, U.K.; ‡ Department of Chemistry, University College London, 20 Gordon Street, London WC1H 0AJ, U.K.; § EaStCHEM School of Chemistry and Biomedical Sciences Research Complex, University of St Andrews, St Andrews, Scotland KY16 9ST, U.K.

## Abstract

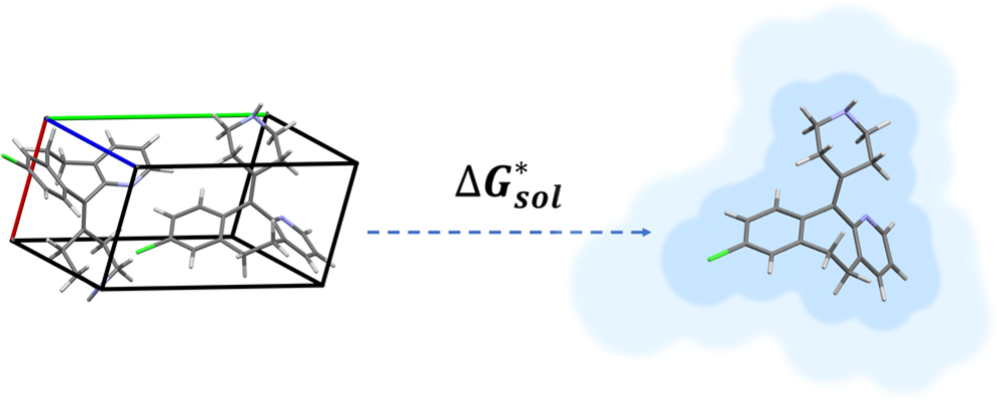

We demonstrate that physics-based calculations of intrinsic aqueous solubility can rival cheminformatics-based machine learning predictions. A proof-of-concept was developed for a physics-based approach via a sublimation thermodynamic cycle, building upon previous work that relied upon several thermodynamic approximations, notably the 2*RT* approximation, and limited conformational sampling. Here, we apply improvements to our sublimation free-energy model with the use of crystal phonon mode calculations to capture the contributions of the vibrational modes of the crystal. Including these improvements with lattice energies computed using the model-potential-based Ψ_mol_ method leads to accurate estimates of sublimation free energy. Combining these with hydration free energies obtained from either molecular dynamics free-energy perturbation simulations or density functional theory calculations, solubilities comparable to both experiment and informatics predictions are obtained. The application to coronene, succinic acid, and the pharmaceutical desloratadine shows how the methods must be adapted for the adoption of different conformations in different phases. The approach has the flexibility to extend to applications that cannot be covered by informatics methods.

## Introduction

Solubility is a fundamental physicochemical property, understanding of which is essential for design and manufacturing processes in industries ranging from petrochemicals to energy materials. It is of particular significance for the pharmaceutical industry, with up to 70% of drugs in development having solubility problems and with low aqueous solubility being a frequent cause of failure of drug candidates.^1,2^ Although the pharmaceutical industry makes extensive use of experimental solubility measurements, they are time-consuming, resource-intensive, and only applicable to already-synthesized molecules, which limits their breadth of application. Consequently, there is a pressing need for accurate computational models to predict solubility.

Recently, various physics-based approaches have been proposed to compute intrinsic aqueous solubility, specifically the equilibrium solubility of the neutral form of the solute, written as *S*_0_. Such methods generally rely on explicit simulations, as with the Frenkel group’s method that identified the nonstandard conditions where the solution has the same chemical potential as the solid.^3,4^ They calculated reversible paths between the Einstein crystal, a simple hypothetical model of a solid, and the real crystalline solute using molecular dynamics (MD) simulations. The aqueous solution phase was modeled with a separate simulation where a cavity was grown in water. A molecule of the solute compound was then placed inside it before the cavity was computationally shrunk to leave the molecule in a simulated aqueous solution. Another method is the direct coexistence approach of Kolafa,^5^ who explicitly simulated a solute dissolving in a solvent and counted the number of solute particles in the simulated solution phase to identify the concentration at which equilibrium was reached. In a strikingly different methodology, the Anwar group used Monte Carlo simulations to compute the density of states of the solution phase. This was designed to produce two separate peaks, one corresponding to the pure solute and the other to the saturated solution. This second peak’s mole fraction of solute was the equilibrium solubility.^6,7^ Lüder and co-workers published four papers aimed at computing the solubility of druglike compounds via simulations.^8−11^ Their studies considered a roundabout route from the solid via amorphous solid and supercooled liquid to the aqueous solution and took advantage of an empirical relationship between the solubilities of the crystalline and amorphous phases, rather than modeling the crystal lattice explicitly. They were able to generate reasonable solubility predictions using only widely affordable simulation techniques but also found that the additional expense of free-energy perturbation (FEP) calculations was rewarded with substantially more accurate results. Mondal et al. have recently also found that free-energy perturbation calculations can successfully model solubility.^12^

The most relevant comparison for the present study is with our own previous work,^13^ which also bears some similarity to the more recent approach of Abramov et al.^14^ We used a sublimation cycle approach, computing the free-energy changes of sublimation and hydration under standard conditions and then summing them to obtain the free energy of solution and hence the equilibrium constant describing aqueous solubility. Those preliminary results showed that solubility can be accurately calculated without empirical parameterization against experimental data, with possible procedural improvements further narrowing the gap between predicted and experimental data. Such improvements are now possible, as we can calculate all of the modes in the crystal using periodic density functional methods, and hence no longer need to use rigid-body crystal modes to estimate the entropy of sublimation. Careful analysis of the vibrational modes enables us to convert lattice energies into sublimation enthalpies without relying on the 2*RT* approximation used in previous work. Improved hydration free energies are computed using two separate approaches: density functional theory (DFT) with full enumeration of low-energy gas and solution-phase conformers, and molecular dynamics (MD) simulations with free-energy perturbation (FEP). It is our belief that such physics-based solubility predictions can rival the currently dominant cheminformatics and machine learning methods with a more physically grounded approach, representing the thermodynamics of each stage of the solubility process.

## Theory

### Calculation of Intrinsic Aqueous Solubility from Solution Free Energy

**Figure 1:**
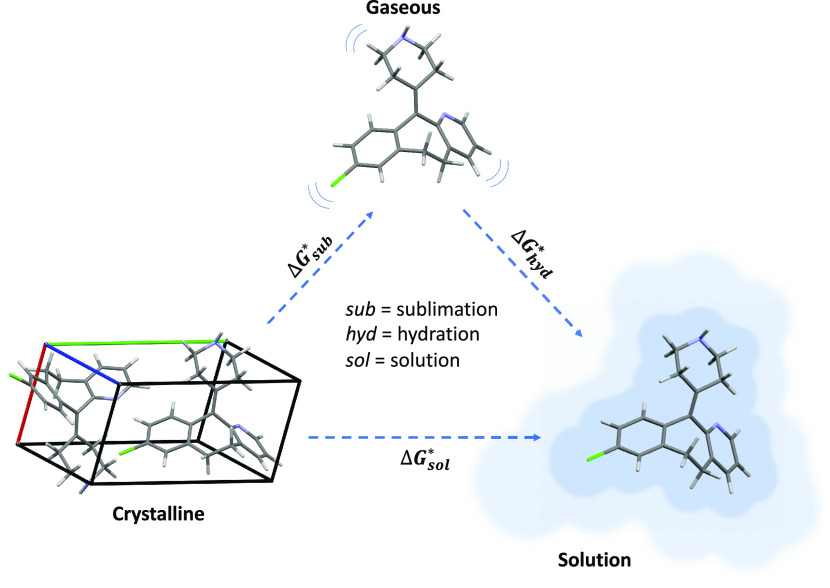
Thermodynamic cycle for transfer from crystal to gas to solution.

Intrinsic aqueous solubility is defined as the concentration of the neutral form of the molecule in a saturated aqueous solution at thermodynamic equilibrium.^15,16^ If the activity coefficient for the solute in solution is assumed to be unity, then the link between intrinsic solubility and Gibbs solution free energy is
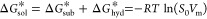
1where Δ*G*_sol_^*^, Δ*G*_sub_^*^, and Δ*G*_hyd_^*^ are the Gibbs free energies for solution, sublimation, and hydration, respectively, *R* is the molar gas constant, *T* is the temperature, *V*_m_ describes the molar volume of the crystal, and *S*_0_ refers to the intrinsic solubility (using moles per liter, mol/L). The superscript asterisk indicates that the Ben-Naim terminology is being used and refers to the Gibbs free energy for transfer of a molecule between two phases at a fixed center of mass in each phase.^17,18^ The relationship between Δ*G*_sol_^*^, Δ*G*_sub_^*^, and Δ*G*_hyd_^*^ is based on a thermodynamic cycle via the gas phase, as illustrated in Figure 1.

Although solvation free energies are commonly reported in the Ben-Naim standard states, sublimation free energies are more commonly calculated and reported relative to a 1 atm standard state in the gas phase, Δ*G*_sub_^0^. The conversion between them is
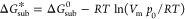
2where *p*_0_ is the atmospheric pressure. Combining eqs 1 and 2 gives an expression for *S*_o_ that does not include *V*_m_
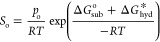
3

### Calculation of Sublimation Free Energy beyond the 2*RT* Approximation

Previous calculations of solubility via the sublimation cycle have relied upon the 2*RT* approximation to convert calculated crystal lattice energies into sublimation enthalpies.^13^ Breaking down this approximation by first ignoring phonon dispersion in the crystal (i.e., considering the Γ-point phonons only) and assuming that the intramolecular vibrations of a molecule are the same in the gas phase and the crystal enable the sublimation enthalpy to be written as
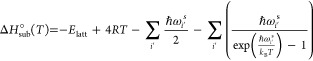
4

The summations run only over the intermolecular phonon modes, which are assumed not to mix with the intramolecular vibrational modes at all, an approximation that is clearly more appropriate to small rigid molecules. If the intermolecular modes were at low frequencies (<200 cm^–1^), then at room temperature, they could be considered as classical harmonic oscillators, i.e., their zero-point energies can be ignored and the equipartition theorem can be applied. There are six such modes for each molecule in the unit cell; thus, the last two terms in eq 4 can be replaced as −6*RT*, and one arrives at the 2*RT* approximation^19^ using eq 5
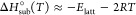
5

However, the 2*RT* approximation is not sufficiently accurate for quantitative solubility predictions. Indeed, recent calculations even on small organic crystals have shown that the approximation inherent in eq 5 can be seriously in error.^20^ When there is intermolecular hydrogen bonding or intramolecular modes that are similar to or even lower in frequency than the lattice phonon modes, the assumptions that the modes do not mix and that their contributions follow equipartition are highly questionable.^20^ Hence, we contend that a clear route to improvement in modeling lattice thermodynamics lies in revisiting the 2*RT* approximation. Through resource-intensive phonon calculations, current periodic DFT-D codes can provide the enthalpy, entropy, and free-energy contributions of each vibrational and phonon mode. We believe that such accurate computation is a prerequisite for the chemical accuracy needed to compute aqueous solubility with an RMS error comparable with that of informatics methods,^21^ around 0.7–1.1 log *S*_0_ units, or the typical experimental error of around 0.6–0.7 log *S*_0_ units.^22^

### Hydration Free-Energy Calculations

Previous predictions of solubility via a sublimation cycle have used implicit solvent models to compute hydration free energy from a single low-energy conformer in each phase. Here, we investigate two alternative approaches that explicitly account for the conformational degrees of freedom of the solute. First, we use density functional theory and a Boltzmann-weighting scheme to compute hydration free energies from an ensemble of low-energy conformers in each phase. Second, we compute hydration free energies from atomistic molecular dynamics simulations using free-energy perturbation methods.

## Computational Methods

### Data Set

**Figure 2:**
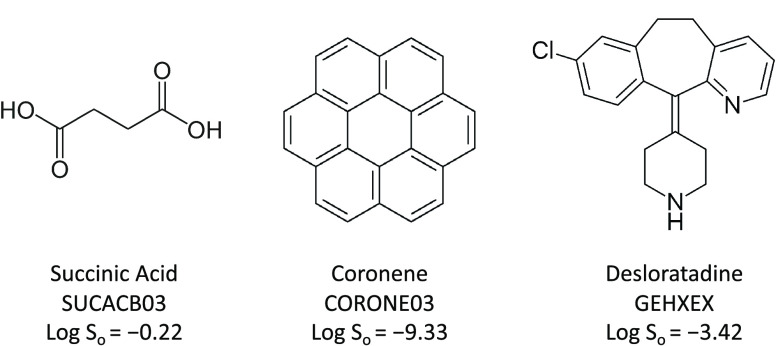
Structures, Cambridge Structural Database refcodes, and experimental solubilities of the three compounds considered in this study.

A small data set of three druglike molecules, succinic acid, coronene, and desloratadine, was used to test these physics-based methods. These three molecules contain differing chemical structures and functional groups, representing a wide range of flexibilities, sizes, and solubilities. All three have multiple polymorphs; however, this study focuses only on the thermodynamically most stable form of each compound under ambient conditions. The chemical structures, common molecular names, and Cambridge Structural Database (CSD) refcodes for the polymorph used in calculations are shown in Figure 2.

Experimental data was found in the literature, with intrinsic aqueous solubilities (measured as mol/L) reported for each molecule as follows: Forbes and Coolidge^23^ reported a value of log *S*_0_ = −0.22 at 25 °C for succinic acid; Miller et al.^24^ reported a value of log *S*_0_ = −9.33 at 25 °C for coronene; Popović et al.^25^ reported a value of log *S*_0_ = −3.42 at 25 °C for desloratadine. Since the crystalline polymorphic forms of the solutes in these solubility assays were not specified, the sublimation calculations were performed using the polymorph of each solute that is known to be most stable under ambient conditions. The sublimation calculations were performed using the β polymorph of succinic acid (CSD^26^ refcode: SUCACB03), the γ polymorph of coronene (CSD refcode: CORONE03), and Form I of desloratadine (CSD refcode: GEHXEX). Further details of the polymorphs and crystal structures of these three compounds are given in the Supporting Information, along with full details of the computational methods and a diagrammatic workflow in Figure S2.

Experimental sublimation enthalpies were found for succinic acid and coronene, reported as 123.2 kJ/mol by Ribeiro da Silva et al.^27^ and 148.2 kJ/mol by Chickos et al.,^28^ respectively. A hydration free energy of −61.08 kJ/mol, measured by Rees and Wolfe,^29^ was found for succinic acid. Where experimental values could not be found, in some cases, it was possible to estimate pseudo-experimental values using available data and standard thermodynamic relationships.

### Calculation of Sublimation Free Energy Using CASTEP and the Model-Potential-Based Ψ_mol_ Method

For all three crystal structures, full DFT-D crystal structure optimizations were carried out with CASTEP^30^ using the Perdew–Burke–Ernzerhof (PBE) functional and the Tkatchenko–Scheffler (TS)^31^ dispersion correction scheme, with on-the-fly pseudopotentials. The input coordinates were the experimental structures (CORONE03, SUCACB03, GEHXEX)^32,33^ in the Cambridge Structural Database with the C–H bond lengths corrected to neutron values.^34^ The optimized crystal structures are in very good agreement with the experimental low-temperature structure determinations (Table S1 in the Supporting Information).

PBE-TS harmonic phonon calculations were performed using either linear response or a finite differencing algorithm with a supercell selected to ensure there were no imaginary frequencies across the phonon Brillouin zone. Once a phonon calculation was completed, the phonon Brillouin zone was further sampled with a finer nuclear Brillouin zone grid and the resultant phonon density of states was integrated to obtain the thermodynamic corrections for the crystal, namely, zero-point energy (*E*_ZPE_^s^), internal energy (*U*_corr_^s^), and Helmholtz free energy (*A*_corr_^s^) of the crystal and the solid-state contribution to *T*Δ*S*_sub_ at 298.15 K. Details of these calculations and their results are given in Tables S2 and S3 of the Supporting Information. The phonon curve of desloratadine is in reasonable agreement with the room-temperature terahertz spectrum.^35^

The molecular conformations were extracted from the optimized crystal structure using NEIGHCRYS.^36^ These were used to obtain molecular energy in its crystal conformation (*E*_mol_in_cryst_) and distributed multipoles (DMA) by GDMA^37^ analysis for use in the lattice energy calculations. Both the PBE/6-311++G(2d,p) and PBE0/6-31G(d,p) charge distributions were obtained using Gaussian 09^38^ with and without a polarizable continuum model (PCM) model (ε = 3.0). The PCM calculations used default settings in Gaussian 09 and a relative dielectric constant of 3.0, typical for organic crystals.^39^ The sensitivity of the results to these four model charge distributions is explored in Table S5 of the Supporting Information, which also shows that the periodic DFT-D lattice energies with the PBE functional are inadequate.

The molecular conformations were optimized using the PBE/6-311++G(2d,p) charge density within the PCM model (ε = 3.0) to the global minimum of the molecule to obtain *E*_mol_min_, and the harmonic vibrational modes are calculated. In the cases of succinic acid and desloratadine, starting from the extracted conformations led to the closest local stationary points on the potential energy surfaces, which were planar and the AAA conformation, respectively. Further optimizations located the global minimum (gauche or SAA), with an energy of *E*_mol_min_. The conformational energy difference between the molecule’s lowest energy gas-phase conformation and the crystal conformation is obtained as the difference between *E*_mol_min_ and *E*_mol_in_cryst_. The molecular vibrations were computed for each gas-phase molecular structure at the global minimum for each compound, with Gaussian 09^37^ with each functional/basis set/PCM combination and “tight” convergence criteria. Thermal analysis by Gaussian 09^37^ yielded *E*_ZPE_^g^, *H*_corr_^g^, and *A*_corr_^g^ for the most stable isolated molecular conformation.

The PBE-TS-optimized crystal structure was reoptimized using DMACRYS^36^ to obtain the intermolecular lattice energy, *U*_inter_, keeping the molecule rigid. The lattice energy was evaluated using the distributed multipoles from the various molecular charge densities (Ψ_mol_-approach^36^) combined with the FIT exp-6 intermolecular repulsion–dispersion pair potential, which has been parameterized by fitting to crystal structures and some heats of sublimation. The lattice energy is then obtained as
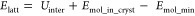
6

Thermodynamic terms calculated above were then combined to obtain Δ*G*_sub_^°^ according to
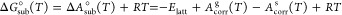
7

### Calculation of Hydration Free Energy Using Implicit Continuum Models

Hydration free energies were calculated using the PBE,^40^ PBE0,^41^ and PBE0-DH^42^ functionals with the 6-311++G(2d,p) basis set and the SMD solvent model.^43^ All hydration calculations were carried out in Gaussian 16.^44^ The PBE functional and basis set were chosen for consistency with the sublimation free-energy calculations, and PBE0 and PBE0-DH were included as potentially more accurate functionals. The SMD solvent model was selected because it performs well for organic molecules.^43^

Three different approaches were investigated to account for conformational degrees of freedom in the calculation of hydration free energy. In the first two approaches, the solute in the gas phase was modeled using the same single conformer as in the sublimation calculations, and the solute in the solution-phase was modeled using either a Boltzmann-weighted ensemble of conformers (SFE1) or a single global minimum energy solution-phase conformer (SFE2). Both of these methods allow for favorable cancellation of errors when the sublimation and hydration legs of the cycle use the same DFT methods. The third approach uses a Boltzmann-weighted ensemble of conformers in each phase separately (SFE3). Conformational searches were carried out using a force-field-based genetic algorithm in OpenBabel^45^ before the low-energy conformers were reoptimized using DFT. Optimized structures were clustered to remove duplicates prior to Boltzmann weighting (see the Supporting Information for further details). Eight dominant solution-phase conformations have previously been identified for desloratadine,^35^ which were used in these calculations. Due to the rigidity of coronene, no conformational search was needed, and a single conformer was used for calculations.

### Calculation of Hydration Free Energy Using Molecular Dynamics Simulations and Free-Energy Perturbation Theory

For each solute, parameters from the general Amber force field (GAFF) with AM1-BCC charges were assigned using the ACPYPE server.^46^ Molecular dynamics simulations were performed using Gromacs 2020.3.^47^ A rhombic dodecahedron box with periodic boundary conditions was used. Water was represented using an SPC/E model,^48^ and no counterions were added. All bonds involving hydrogen were kept rigid using the LINCS algorithm of the fourth order. Dynamics were simulated using a stochastic dynamics integrator, with a reference temperature of 298K. Neighbor searching was performed using a pair list generated by a Verlet cutoff scheme. Short-range interactions used the particle-mesh Ewald (PME) method,^49^ with Lennard-Jones interactions switched off at 10 Å. Electrostatic interactions were treated using the PME method with a cutoff of 10 Å, a Fourier spacing of 1.2 Å, a fourth-order interpolation, and a tolerance of 10^–4^.

Hydration free energy was computed using 21 values of the scaling factor λ, with Lennard-Jones and electrostatics interactions between the solute and solvent scaled together. Intramolecular interactions were kept the same at all λ values. Calculations were performed at 21 λ values at intervals of 0.05 from 0 to 1. Each simulation with its corresponding λ ran for 1300 ps during its production run. Prior to running a production MD simulation, 2500 steps of steepest descent optimization and a 50 ps equilibration were performed. A time step of 2 fs was used for each simulation. In both equilibration and production runs, the pressure was kept constant at 1 bar using the Parrinello–Rahman pressure coupling^50^ and a compressibility of 4.5 × 10^–5^ bar^–1^. After each simulation was complete, hydration free energy was evaluated using the Bennett acceptance ratio (BAR).^51^

### Prediction of Intrinsic Aqueous Solubility Using Machine Learning Algorithms

Three machine learning models were implemented for predicting the intrinsic solubility of succinic acid, desloratadine, and coronene. These models used the Extra Trees,^52^ Random Forest,^53^ and Bagging^54^ algorithms, each trained on a set of 117 druglike compounds with 173 CDK descriptors^55^ used for each molecule and implemented as described in reference (56). The training set for these ML models does not include succinic acid, desloratadine, or coronene. These models were initially developed for an entry to the 2019 Solubility Challenge.^56^

## Results

### Sublimation Free Energy

In Table 1, we present sublimation thermodynamics computed using the DMACRYS PBE/6-311++G(2d,p)/PCM Ψ_mol_-based lattice energies and the thermal corrections computed from periodic PBE-TS phonons, as described in the Computational Methods section and elaborated on in the Supporting Information. The calculated sublimation enthalpies are in good agreement with the measured values for succinic acid (Δ*H*_sub_^°expt^ – Δ*H*_sub_^°calcd^ = 2.16 kJ/mol) and coronene (Δ*H*_sub_^°expt^ – Δ*H*_sub_^°calcd^ = 4.69 kJ/mol); no experimental sublimation data was available for desloratadine. These errors would correspond to 2.4- and 6.6-fold errors in solubility (based on eq 1), respectively, which is encouraging given that machine learning models commonly report 10-fold errors. The influence of using crystal phonon modes rather than the 2*RT* approximation to convert lattice energies into sublimation enthalpies can be assessed by considering the value of the term Δ*H*_sub_^°^ + *E*_latt_ in Table 1. Although there is good agreement between Δ*H*_sub_^°calcd^ + *E*_latt_ and −4.96 kJ/mol (=–2*RT*) for succinic acid, for coronene and desloratadine, the differences would correspond to more than 17- and 10-fold differences in solubility, respectively. Clearly, the method used to convert lattice energy into sublimation enthalpy has a large effect on predicted solubility.

**Table 1:** Lattice and Sublimation Energetics in kJ/mol Based on DMACRYS PBE/6-311++G(2d,p)/PCM Calculations and Thermal Corrections^a^

compound	*E*_latt_	Δ*H*_sub_^°calcd^	Δ*H*_sub_^°expt^	Δ*H*_sub_^°^	Δ*G*_sub_^°^	*T*Δ*H*_sub_^°^	Δ*H*_sub_^°calcd^ + *E*_latt_
succinic acid	–125.89	121.04	123.2	49.06	51.54	69.50	–4.85
coronene	–155.61	143.51	148.2	76.57	79.05	64.46	–12.10
desloratadine	–144.40	133.72		57.29	59.77	73.95	–10.68

a The experimental Δ*H*_sub_^°expt^ is given where available.^27,28^

### Hydration Free Energy

Table 2 reports hydration free energies computed using atomistic MD/FEP simulations and three DFT methods (PBE/6-311++G(2d,p)/SMD, PBE0/6-311++G(2d,p)/SMD, PBE0-DH/6-311++G(2d,p)/SMD). For succinic acid and coronene, for which experimental or pseudo-experimental values are available, MD/FEP is the most accurate method for computing hydration free energy and gives relatively small errors (Δ*G*_hyd_^expt^ – Δ*G*_hyd_^calcd^ of −3.61 and 1.6 kJ/mol, respectively). For the DFT methods, taking a Boltzmann-weighted average of multiple conformers, rather than a single minimum energy conformer in the gas and/or solution phase, had relatively little effect on the results, leading to small changes in Δ*G*_hyd_ for succinic acid in most cases (Table S8 in the Supporting Information). Slightly larger changes were observed for desloratadine when using the PBE0 or PBE0-DH functionals (ΔΔ*G*_hyd_ < 2 kJ/mol), but there was no evidence that the Boltzmann-weighting scheme led to more accurate results overall. For that reason, Table 2 presents SMD results obtained by the SFE2 approach only. However, for all solutes, changing from PBE to PBE0 or PBE0-DH functionals led to a non-negligible change in hydration free energy. For succinic acid, PBE0-DH agrees reasonably well with experiment (Δ*G*_hyd_^expt^ – Δ*G*_hyd_^calcd^ =–4.85 kJ/mol), whereas PBE does not (Δ*G*_hyd_^expt^ – Δ*G*_hyd_^calcd^ = −11.75 kJ/mol). A similar trend is observed for coronene although neither PBE nor PBE0-DH gives satisfactory results. For desloratadine, the DFT and MD/FEP methodologies give self-consistent results, but there is no experimental data with which to compare them.

**Table 2:** Hydration Free Energies from Experiment and Computed from DFT or MD/FEP Simulations Using the SFE2 Approach^a^

compound	hydration model	Δ*G*_hyd_^*calcd^ (kJ/mol)	Δ*G*_hyd_^*expt^ (kJ/mol)
succinic acid	PBE/6-311++G(2d,p)/SMD	–49.33	–61.08
	PBE0/6-311++G(2d,p)/SMD	–52.78	
	PBE0-DH/6-311++G(2d,p)/SMD	–56.23	
	GAFF/AM1-BCC, SPC/E	–57.47	
coronene	PBE/6-311++G(2d,p)/SMD	–18.68	–38.40
	PBE0/6-311++G(2d,p)/SMD	–23.01	
	PBE0-DH/6-311++G(2d,p)/SMD	–26.32	
	GAFF/AM1-BCC, SPC/E	–40.00	
desloratadine	PBE/6-311++G(2d,p)/SMD	–45.11	
	PBE0/6-311++G(2d,p)/SMD	–48.08	
	PBE0-DH/6-311++G(2d,p)/SMD	–50.38	
	GAFF/AM1–BCC, SPC/E	–44.93	

a The experimental Δ*G*_hyd_^*expt^ is given where available.^29^ While we do not have a true experimental hydration free energy for coronene, we can infer its value if we assume that the experimental log *S*_0_ and Δ*H*_sub_^°^ values^24,28^ and the computed *T*Δ*S*_sub_^°^ are correct. Rearranging eq 3 then leads to a back-calculated pseudo-experimental Δ*G*_hyd_^*^ of −38.40 kJ/mol.

### Machine Learning Intrinsic Solubility Predictions

Table 3 reports intrinsic solubility predictions from the Extra Trees, Random Forest, and Bagging algorithms. The Extra Trees model performed better than the Random Forest and Bagging models for our data set and was chosen as the benchmark for the physics-based models. The same model was submitted to the 2019 Solubility Challenge by one of us and known for the purposes of that Challenge as JMSA_B.^53,57,58^

**Table 3:** Predicted Log *S*_0_ Values Derived from Machine Learning Extra Trees, Random Forest, and Bagging Algorithms

		log *S*_0_^calcd^		
compound	log *S*_0_^expt^	extra trees	random forest	bagging
succinic acid	–0.22	0.05	–1.00	–1.21
coronene	–9.33	–8.05	–7.35	–6.22
desloratadine	–3.42	–4.30	–4.20	–3.96

### Physics-Based Intrinsic Solubility Predictions

The computed Δ*G*_sub_^°^ and Δ*G*_hyd_^*^ combine according to eq 3 to give the log *S*_0_ values reported in Table 4. The computed log *S*_0_ values are based on DMACRYS PBE/6-311++G(2d,p)/PCM calculations with thermal corrections as the sublimation method and PBE/6-311++G(2d,p)/SMD, PBE0/6-311++G(2d,p)/SMD, PBE0-DH/6-311++G(2d,p)/SMD, or MD/FEP as the hydration method.

**Table 4:** Computed Physics-Based Log *S*_0_ Values Derived from Hydration Free Energy Results Obtained by PBE/6-311++G(2d,p)/SMD, PBE0/6-311++G(2d,p)/SMD, and PBE0-DH/6-311++G(2d,p)/SMD Calculations and MD/FEP Simulations

compound	sublimation model	hydration model	log *S*_0_^calcd^	log *S*_0_^expt^	error
succinic acid	PBE/6-311++G(2d,p)/PCM	PBE/6-311++G(2d,p)/SMD	–1.78	–0.22	1.56
		PBE0/6-311++G(2d,p)/SMD	–1.17		0.95
		PBE0-DH/6-311++G(2d,p)/SMD	–0.57		0.35
		GAFF/AM1-BCC, SPC/E	–0.35		0.13
	extra trees		0.05		–0.27
coronene	PBE/6-311++G(2d,p)/PCM	PBE/6-311++G(2d,p)/SMD	–11.97	–9.33	2.64
		PBE0/6-311++G(2d,p)/SMD	–11.21		1.88
		PBE0-DH/6-311++G(2d,p)/SMD	–10.63		1.30
		GAFF/AM1-BCC, SPC/E	–8.23		–1.10
	extra trees		–8.05		–1.28
desloratadine	PBE/6-311++G(2d,p)/PCM	PBE/6-311++G(2d,p)/SMD	–3.96	–3.42	0.54
		PBE0/6-311++G(2d,p)/SMD	–3.44		0.02
		PBE0-DH/6-311++G(2d,p)/SMD	–3.03		–0.39
		GAFF/AM1-BCC, SPC/E	–3.99		0.57
	extra trees		–4.30		0.88

For succinic acid, PBE/6-311++G(2d,p)/SMD underestimated the magnitude of the hydration free energy, which resulted in an underestimation of the solubility of 1.56 log *S*_0_ units. Replacing PBE by the PBE0-DH functional improved the calculated hydration free energy and log *S*_0_ to within −4.85 kJ/mol and 0.35 log units of their experimental results, respectively. The MD/FEP calculations gave the most accurate hydration free energy and as a result predicted log *S*_0_ = −0.35, only 0.13 log units from the experimental value.

For coronene, using the PBE/6-311++G(2d,p)/SMD hydration model again leads to underestimation of the magnitude of the hydration free energy, with an error of 19.76 kJ/mol as compared to the back-calculated pseudo-experimental Δ*G*_hyd_^*^. This results in a prediction of log *S*_0_ lower than the experimental^24^ value by 2.64 units. (The derivation of our back-calculated pseudo-experimental Δ*G*_hyd_^*^ is described in the footnote of Table 2.) Using instead the PBE0-DH/6-311++G(2d,p)/SMD hydration model gives a more accurate value of Δ*G*_hyd_^calcd^ and leads to a predicted log *S*_0_ of only 1.30 log units below the experimental value, as shown in Table 4. The most accurate hydration free energy was obtained from the MD/FEP simulations, which overestimated the magnitude of Δ*G*_hyd_^calcd^ by only 1.6 kJ/mol compared to the pseudo-experimental value, and resulted in a prediction of solubility within 1.10 log units of the experimental value.

The PBE/6-311++G(2d,p)/SMD hydration model appears to perform better for desloratadine than for the other solutes and gives a calculated log *S*_0_ within 0.54 log *S*_0_ units of the experimental value.^25^ Since we have no experimental sublimation or hydration thermodynamics data, however, it is unclear whether our predictions of Δ*G*_sub_^°^ and Δ*G*_hyd_^*^ are both accurate or whether we are relying on a cancellation of errors to arrive at an accurate log *S*_0_ prediction. The underestimation of solubility suggests that the PBE/6-311++G(2d,p)/SMD hydration model underestimates the magnitude of the hydration free energy, which would be in keeping with the trend observed for coronene and succinic acid, but cannot be independently validated with the available experimental data. Using the PBE0 or PBE0-DH functionals rather than the PBE functional leads to more accurate estimates of solubility, within 0.02 log units and 0.39 log units of the experimental value, respectively. Using the solvation free energy computed by MD/FEP simulations gives a predicted solubility that is almost identical to that obtained using the PBE/6-311++G(2d,p)/SMD model, with an error compared to experiment of 0.57 log units. For desloratadine, all three solvent models give predictions with errors <0.6 log *S*_0_ units, which compares favorably with the Extra Trees regressor that gives an error of 0.88 log *S*_0_ units.

**Figure 3:**
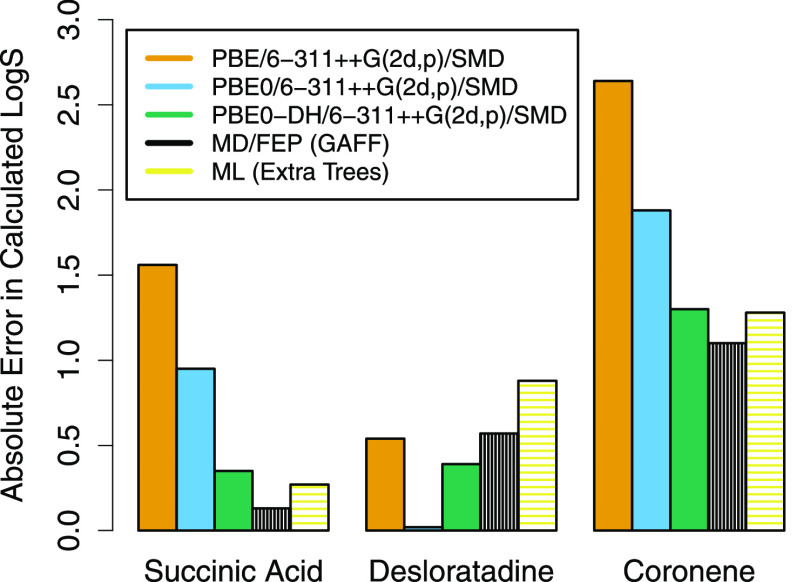
Absolute error in calculated log *S*_0_ for succinic acid, desloratadine, and coronene. The physics-based predictions of solubility use the PBE/6-311++G(2d,p)/PCM sublimation free energies and the PBE/6-311++G(2d,p)/SMD (orange), PBE0/6-311++G(2d,p)/SMD (blue), PBE0-DH/6-311++G(2d,p)/SMD (green), or MD/FEP (black) hydration free energies. The machine learning predictions use the Extra Trees algorithm (yellow).

The absolute error in log *S*_0_ for each model is summarized in Figure 3. The Extra Trees results (yellow bars) provide an example of a state-of-the-art machine learning model against which the physics-based models have been evaluated. Using the DMACRYS PBE/6-311++G(2d,p)/PCM Ψ_mol_-based sublimation method, and either PBE0-DH/6-311++G(2d,p)/SMD or MD/FEP hydration calculations, we obtained predicted solubilities that rival the accuracy of the Extra Trees model.

## Discussion

Our previous physics-based solubility prediction work^13^ gave an RMSE of 1.45 log *S*_0_ units over 25 druglike compounds while incorporating the 2*RT* approximation. Although that was a promising result, further improvements were required to match the predictive accuracy of machine learning. The present work achieves this aim by improving upon the 2*RT* approximation for enthalpies of sublimation by utilizing a full analysis of the vibrational and phonon contributions to the sublimation enthalpy and entropy. Succinic acid and desloratadine adopt different conformations in the solid from the isolated molecule at low temperatures and the ensemble in the liquid. Fortunately, our results suggest that the hydration results are rather insensitive to conformational averaging, and so the conformational search could be limited, provided that the global minimum (gas-phase) conformation and the crystal conformation are known.

We have used the experimental crystal structures in these calculations, but this could be obtained from a crystal structure prediction study.^57^ Indeed, we envisage that these physics-based solubility calculations would be performed alongside such a crystal structure prediction study as these are now becoming more routine in the industry^58^ and being developed to be used during early drug development^59^ at the solid form selection stage and as a complement to solid form screening.^60,61^ The key advantage of a physics-based approach over informatics would then be realized, by being able to adapt the calculations to different solvents, polymorphs, and temperatures.

The linking of the solubility calculations into a workflow involving crystal structure prediction, which includes determining the range of conformations that can occur in solid state, means that the development of this approach to solubility prediction can be closely coupled to the current work on improving the calculation of free energies of polymorphs. Absolute lattice energies calculated using periodic DFT-D and currently affordable functionals like PBE are known to be poor,^62^ but the progress in developing reliable calculations of relative energies of polymorphs^63^ and sublimation pressures^64^ suggests that a fully quantum-mechanical prediction of the solid-state contributions^14,65^ could provide accurate solubilities. This may need to be coupled with the use of higher-level calculations on the isolated molecule, as this has been found to provide a major improvement in CSP results in certain cases of conformational polymorphism.^66,67^ However, methods of mitigating the expense of the phonon calculations, which appear necessary given the inadequacy of the 2*RT* approximation, are being developed.^68^

Alongside developing absolute solubility calculations, it is also to estimate the solubility difference between polymorphs or between racemic and enantiopure crystals. The degree of cancellation of errors is very specific to the crystals involved^20^ and needs to be highly accurate as the average difference in molar solubility between polymorphs has been estimated to be approximately 2-fold,^69^ which is 4–5 times smaller than the average error in solubility models. One outcome of our study is that care has to be taken to ensure cancellation of errors when calculating absolute sublimation free energies, i.e., at this stage, it is more accurate to use consistent electronic structure methods than the best affordable for each phase. It also appears that the hydration energies improve with the electronic structure method used.

In this study, we have chosen three diverse molecules spanning a wide range of solubilities and the results are extremely encouraging. For all three solutes, the implicit solvation model improves with the quality of the molecular charge distribution and is relatively insensitive to the treatment of the conformational flexibility. The explicit solvation model using molecular dynamics simulations provides very worthwhile results, which are capable of reflecting the effects of long-lived, specific hydrogen bonding of solvent to solute, though such extended residence times do not occur in these three systems.^34^ These calculations will depend critically on the quality of the force field, as do many other molecular dynamics-based methods.^70^

Physics-based solubility approaches including the one presented here are typically tested on only a handful of compounds at best. In this case, the three compounds chosen present different types of chemistries, conformational flexibilities, and solubilities. Critically, the set includes desloratadine as a more typical pharmaceutical, showing that the methodology can be applied to larger, flexible molecules than are typically used to validate physics-based methods. In comparison, machine learning and QSPR models are typically validated on tens-to-hundreds of compounds, the two solubility challenges each having a 100-compound test set.^58,71^ A major limitation of informatics approaches is that they can only be applied to properties for which training data for sufficient compounds has been measured. Physics-based approaches have the potential to be modified for different solvent mixtures,^43,72,73^ temperatures,^74,75^ and other properties,^76^ vastly extending the possible contribution of digital design to crystallization processes. Following the proof-of-concept results presented here, the validation of physics-based solubility methods on a larger range of molecules is a priority to drive progress in this field.

## Conclusions

The physics-based method presented within this work shows that intrinsic aqueous solubility can be predicted with reasonable accuracy, rivaling current cheminformatics and machine learning approaches. Throughout this process, a full computational description of each thermodynamic stage of transferring a molecule from crystal to gas to solution is produced. Further progress can be made, however, including systematic improvements to the sublimation and hydration free-energy models, as well as more rigorous testing on a larger data set of druglike molecules.

## Supporting Information Available

Full specification of the theoretical derivations, methodology, and more detailed results including; the workflow developed in this work for physics-based computation of sublimation and hydration (Figure S1), variations in solid and gaseous energies with functional (Tables S5 and S6); tests of hydration free energy methods using the Minnesota Solvation Database (Figures S2 and S3); variation of calculated hydration energies with treatment of conformational variations (Table S8); calculated solubilities from all combinations of methods (Tables S9 and S10) (PDF)
