# Supplementary material for: Toward Physics-Based Solubility Computation for Pharmaceuticals to Rival Informatics
Source: J Chem Theory Comput. 2021 May 14;17(6):3700–9. doi: 10.1021/acs.jctc.1c00130 (PMC8190954; doi:10.1021/acs.jctc.1c00130)
Supplement: Supplementary file 1 — ct1c00130_si_001.pdf [file ct1c00130_si_001.pdf]

Supporting Information for "Toward Physics-Based Solubility Computation for Pharmaceuticals to Rival Informatics"

Daniel J. Fowles,[1] David S. Palmer,[1] Rui Guo,[2] Sarah L. Price,[2] John B. O. Mitchell [3]

[1] Department of Pure and Applied Chemistry, University of Strathclyde, Thomas Graham Building, 295 Cathedral Street, Glasgow, Scotland G1 1XL, U.K.

[2] Department of Chemistry, University College London, 20 Gordon Street, London WC1H 0AJ, U.K.

[3] EaStCHEM School of Chemistry and Biomedical Sciences Research Complex, University of St Andrews, St Andrews, Scotland KY16 9ST, U.K.

Table of Contents

Experimental Data 2
Sublimation Free Energy Calculations 3
I. Theory 3
A. Theoretical basis of the calculation of thermodynamics of sublimation 3
B. Beyond the 2RT Approximation 4
C. Standard State for Sublimation Thermodynamics 4
II. Methods 5
A. Protocol for first principles computation of sublimation free energy 5
B. Crystal structure optimisation 6
C. Phonon calculations and thermodynamic corrections for the crystals 7
D. Lattice energies 8
1. Periodic DFT-D lattice energies 8
2. Lattice energies using model intermolecular potentials and the Ψ_{mol} approach 9
3. Choice of lattice energy model 11
E. Thermodynamic corrections for gas-phase molecules 11
III. Results 12
A. Thermodynamics of sublimation 12
Solvation Free Energy Calculations 14
I. Methods 14

A. Additional Information about the Clustering Method used in the Calculation of SFE1 and SFE3. 14
B. Comment on Thermal Corrections to the Solvation Free Energy 14
II. Results 15
A. Minnesota Solvation Database 15
B. Succinic Acid, Desloratadine, and Coronene 16
Solubility Prediction from Sublimation and Hydration Free Energies 18

Experimental Data

The three molecules, coronene, succinic acid and desloratadine, show a range of aqueous solubilities, and a progression in size and conformational flexibility. All three have multiple polymorphs, however this study focuses on the thermodynamically most stable form of each molecule at room temperature. Details of the experimental crystal structures of the three molecules can be found in Table S1.

Succinic acid (CSD[1] REFCODE: SUCACB03) has three polymorphs, but the α form is highly metastable and the conformational γ polymorph has only been observed once.[2] Succinic acid in the β form exists in the extended conformation with all four carbon atoms in one plane, which has been calculated to be slightly higher in energy than the gauche conformation in the γ form. The gauche conformer is the dominant conformation in solution. The phonon and relative stability calculations suggest that γ may be the most stable form at low temperatures, but the β form is the most stable form under ambient conditions. The experimental solubility of $\log S_0$ = -0.22 at 25°C was measured by Forbes & Coolidge.[3] The sublimation enthalpy was reported as 123.2 kJ/mol by Ribeiro da Silva *et al.*[4] The form was not specified, but this solubility is expected to be that of the β form. The hydration free energy was reported by Rees & Wolfe[5] as -61.08 kJ/mol.

The γ polymorph of **coronene** (CSD REFCODE: CORONE03), a rigid molecule, is the most stable polymorph at room temperature. The β polymorph has only recently been discovered by crystallisation in a magnetic field and was shown to be more stable at low temperature, though the two polymorphs are closely related,[6,7] with a 1st order thermal phase transition between them. All the experimental thermodynamic and solubility measurements are for the γ polymorph. The measured solubility is $\log S_0$ = -9.33 at 25°C by Miller *et al.*[8] The sublimation enthalpy was measured as 148.2 kJ/mol by Chickos *et al.*[9]

Form I of **desloratadine** (CSD REFCODE: GEHXEX) exhibits two endothermic transitions upon heating with peak temperatures of 338.6 K and 431.3 K, corresponding to successive transitions to form II and form III.[10] These transitions are reversible, and so any 298 K thermodynamic data and solubility data will be that of form I. The $P2_1$ form I has been determined in reference[10] to have an experimental cell volume of 773.8(5)Å^3 at 293 K and 755.2(2)Å^3 at 80 K. The phonon curves are in reasonable agreement with the room-temperature terahertz spectrum.[10] Desloratadine in form I is in the AAA conformation, one of the eight conformational minima, four of which are more than 3 kJ/mol higher (PBE0/6-31G(d,p)), while the other three are about 1 kJ/mol in conformational energy above the global minimum, the SAA conformation. The experimental intrinsic aqueous solubility obtained by Popovic *et al.* is $\log S_0$ = -3.42 at 25°C.[11]

Sublimation Free Energy Calculations

I. Theory

A. Theoretical basis of the calculation of thermodynamics of sublimation

All thermodynamic properties and equations in this section are assumed to be under the standard states of 1 atmosphere pressure at 298.15K (see section I.C), e.g. ΔH°_{sub} etc. For the sake of clarity, the superscript ° has been omitted in this section.

The enthalpy of sublimation ΔH_{sub} can be expressed as:

$$\Delta H_{sub} = -E_{latt} + \left(E^{g}_{ZPE} - E^{s}_{ZPE}\right) + \int_{T'=0}^{T}(C^{g}_{p}(T') - C^{s}_{p}(T'))dT' \quad (1)$$

where E_{latt} is the crystal lattice energy, E_{ZPE} is the zero point energy, C_p is the constant-pressure heat capacity, T is the temperature, and superscripts g and s refer to the gas and solid (crystalline) phases, respectively. Since $E_{latt} = E^{s}_{elec} - E^{g}_{elec}$, it has the opposite sign from sublimation enthalpy and is negative. The entropy of sublimation ΔS_{sub} can be written as:

$$T\Delta S_{sub} = T\int_{T'=0}^{T}(C^{g}_{p}(T')/T' - C^{s}_{p}(T')/T')dT'. \quad (2)$$

Making the approximation that the pressure-volume work (pV term) for the solid can be neglected, the enthalpy of sublimation ΔH_{sub} of a crystal to the gas phase can also be expressed as:

$$\Delta H_{sub}(T) = H^{g}(T) - H^{s}(T) = H^{g}(T) - U^{s}(T). \quad (3)$$

Defined in this way, $\Delta H_{sub}(T)$ is always positive. The internal energy of the solid phase $U^{s}(T)$ can be calculated from the total electronic energy of the solid E^{s}_{elec} plus an addition correction $U^{s}_{corr}(T)$ that can be computed with phonons from CASTEP[12] :

$$U^{s}(T) = E^{s}_{elec} + U^{s}_{corr}(T) = E^{s}_{elec} + E^{s}_{ZPE} + \sum_{k,i}\left(\frac{\hbar\omega^{s}_{k,i}}{\exp\left(\frac{\hbar\omega^{s}_{k,i}}{k_B T}\right)-1}\right), \quad (4)$$

where the last term, involving the sum of the i normal modes with frequency $\omega^{s}_{k,i}$ over the k points in the Brillouin zone, represents $\int_{T'=0}^{T} C^{s}_{v}(T')dT'$.

In the gas-phase, $H^{g}(T)$ was also calculated as the sum of the gas-phase energy E^{g}_{elec} and an additional correction term $H^{g}_{corr}(T)$:

$$H^{g}(T) = E^{g}_{elec} + H^{g}_{corr}(T). \quad (5)$$

Treating the molecules as non-interacting in the gas-phase, H^{g}_{corr} can be calculated as the sum of contributions from the pV term (RT), the translation ($3RT/2$), rotation (in general $3RT/2$) and vibrational modes of the molecule. This can be explicitly written as:

$$H^{g}_{corr}(T) = 4RT + E^{g}_{ZPE} + \sum_{j}\frac{\hbar\omega^{g}_{j}}{\exp\left(\frac{\hbar\omega^{g}_{j}}{k_B T}\right)-1}, \quad (6)$$

with the sum again over j (intramolecular) vibrational normal modes of the molecule, and $E^{g}_{ZPE} = \sum_{j}\hbar\omega^{g}_{j}/2$. Note the ZPEs are already included in $H^{g}_{corr}(T)$ and $U^{s}_{corr}(T)$ in normal Gaussian09[13] and CASTEP[12] output. Thus:

$$\Delta H_{sub}(T) = -E_{latt} + H^{g}_{corr}(T) - U^{s}_{corr}(T) \quad (7)$$

The Helmholtz free energy change of sublimation can be calculated as:

$$\Delta A_{sub}(T) = -E_{latt} + A^{g}_{corr}(T) - A^{s}_{corr}(T); \quad (8)$$

Again ignoring the pV term in the solid phase, and assuming an ideal gas for the gas phase, the Gibbs free energy change of sublimation is:

$$\Delta G_{sub}(T) = \Delta A_{sub}(T) + RT = -E_{latt} + A^{g}_{corr}(T) - A^{s}_{corr}(T) + RT \quad (9)$$

and $T\Delta S_{sub} = \Delta H_{sub}(T) - \Delta G_{sub}(T)$.

The lattice energy E_{latt} is the main component of ΔH_{sub}, though the two are defined so that they have opposite signs.

B. Beyond the 2RT Approximation

The additional components of ΔH°_{sub} have often been estimated by the 2RT approximation: [14,15,16]

$$\Delta H^{\circ}_{sub}(T) = -E_{latt} - 2RT, \quad (10)$$

However recent calculations on small organic crystals have shown that this 2RT approximation can be seriously in error.[14]

In the 2RT approximation one assumes, amongst other things, that intramolecular vibrational modes are not affected by the crystal packing even when there is intermolecular hydrogen bonding, that intramolecular modes which are similar to or even lower in frequency than the lattice phonon modes do not mix with those intermolecular modes in the crystalline phase, and that the phonon modes' contributions follow equipartition. The first two of these, at least, are highly questionable.[14] Hence, a clear route to improvement in modelling lattice thermodynamics lies in revisiting the 2RT approximation.

Through resource-intensive phonon calculations, current periodic DFT-D codes, *e.g.*, CASTEP,[13] can provide the thermodynamic corrections (U^{s}_{corr} and A^{s}_{corr}) for the crystal. Standard quantum chemical software applications such as Gaussian09[13] can give the corresponding thermodynamic correction terms (H^{g}_{corr} and A^{g}_{corr}) of isolated gas phase molecules. Using Equations (7), (8) and (9), which needs the absolute value of lattice energy, the thermodynamic properties associated with sublimation can be calculated. Thus, we obtain the enthalpy, entropy and free energy contributions of each vibrational and phonon mode. We believe that these accurate computations, explicitly considering vibrational and phonon modes in the gas and solid phases, are a prerequisite for the chemical accuracy needed to compute aqueous solubility to an accuracy comparable with either that of informatics methods, around 0.7 to 1.1 $\log S_0$ units, or the typical experimental error of around 0.6 to 0.7 $\log S_0$ units.[17] On the other hand, the calculation of the absolute lattice energy of an organic crystal is by no means trivial.

C. Standard State for Sublimation Thermodynamics

In the above, we calculate pV on the assumption of 1 atmosphere pressure at 298.15K. Hence, we are using the 1 atmosphere standard state when calculating thermodynamic quantities associated with sublimation. Thus, the enthalpy, entropy and Gibbs free energy of sublimation calculated are, using the nomenclature of reference[18], ΔH°_{sub}, ΔS°_{sub}, and ΔG°_{sub}, where the symbol ° indicates the use of a 1 atmosphere standard state.

II. Methods

A. Protocol for first principles computation of sublimation free energy

The protocols adopted in this work are listed below, summarized in Figure S1 and given in detail as the results for each step are analysed:

1) The experimental crystal structure is optimised with CASTEP using PBE-TS, and the harmonic phonon spectra calculated. From calculated crystal phonons, E^{s}_{ZPE}, U^{s}_{corr}, A^{s}_{corr} and the solid state contribution to $T\Delta S_{sub}$ at 298.15 K are calculated.
2) The molecular conformation is extracted from the optimised crystal structure in step 1), using NEIGHCRYS.[19] The molecular energy in its crystal conformation ($E_{mol\_in\_cryst}$) and distributed multipoles (DMA) are evaluated for the PBE/6-311++G(2d,p) charge density using a PCM model with ε=3.
3) The molecular conformation is optimised using the PBE/6-311++G(2d,p) charge density within the PCM model (ε=3) to the global minimum of the molecule to obtain the E_{mol_min}, and the harmonic vibrational modes are calculated. This also provides E^{g}_{ZPE}, H^{g}_{corr}, A^{g}_{corr} and a gas-phase molecular structure at the global minimum.
4) The PCM-optimized molecular structure is used to compute hydration free energy by the four methods described in the article: (i) Molecular Dynamics simulations using GAFF combined with Free Energy Perturbation; (ii-iv) three different density functionals (PBE, PBE0, PBE0-DH) combined with the 6-311++G(2d,p) basis set and SMD implicit continuum model for water.
5) The PBE-TS optimized crystal structure in step (1) is re-optimised using DMACRYS to obtain the intermolecular interaction energy, U_{inter}, while keeping the molecule rigid, and using an intermolecular potential calculated from the distributed multipoles from step (2) and the FIT *exp-6* repulsion-dispersion model. The lattice energy can then be obtained as: $E_{latt} = U_{inter} + E_{mol\_in\_cryst} - E_{mol\_min}$.
6) The components of the thermodynamic cycle are combined to estimate the intrinsic aqueous solubility according to the equation:[18]

$$S_0 = \frac{p_0}{RT} exp\left(\frac{\Delta G^{\circ}_{sub} + \Delta G^{*}_{hydr}}{-RT}\right) \quad (12)$$

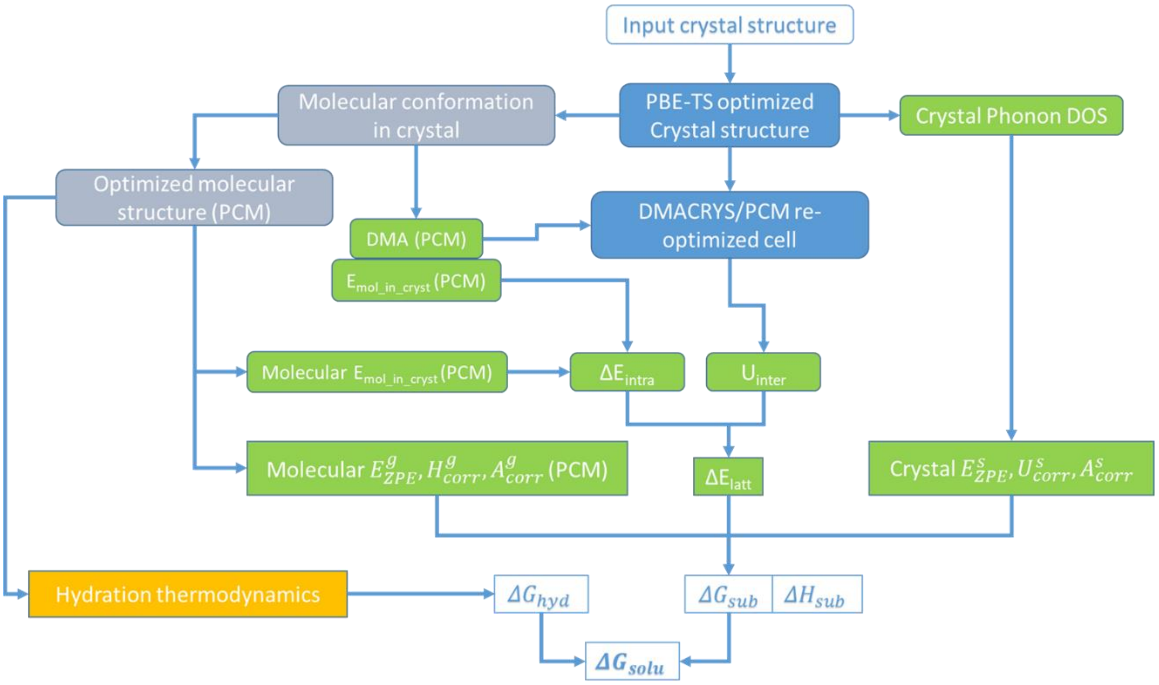

Figure S1 The protocol developed in this work for physics-based computation of sublimation and hydration free energies and their application to solubility calculations.

B. Crystal structure optimisation

For all three crystal structures, full DFT-D crystal structure optimizations were carried out with CASTEP[12] using the PBE functional and Tkatchenko-Scheffler (TS)[20] dispersion correction scheme, with on-the-fly pseudopotentials. Initial structures for the optimizations came from published searches for crystal structure prediction for succinic acid[2] and desloratadine,[10] where the molecular conformation in the crystal is AAA. For coronene, the initial structure was taken from the experimental structure (REFCODE: CORONE03)[6,7] in the Cambridge Structural Database with the C-H bond lengths corrected to neutron values. For each structure, a plane wave cut-off energy and Monkhorst-Pack k-point grid spacing[21] were selected after extensive convergence testing. The PBE-TS optimization was carried out using the BFGS (Broyden-Fletcher-Golfarb-Shanno) algorithm with an SCF electronic energy tolerance of 10^{-10} eV, force convergence tolerance of 0.001 eV/Å, and fine grid scale of 4. Cell parameters of the PBE-TS optimized crystal structures are listed in Table S1, and compared to those of the experimental ones. Details of parameters used in the optimizations are summarised in Table S2. More details of PBE-TS structure optimization can be found in reference 10.

Although the PBE functional has been recently shown to fail to reproduce the structure of an organic polymorph,[22] the next level on the Jacob's ladder of density functionals, i.e., hybrid functionals, are prohibitively costly in computing resource for all but a few crystal structures. This choice of density functional has considerable impact on the adopted approach for the calculation of lattice energy in Section II.D.

Table S1 The polymorphs selected for the solubility calculations. Cell parameters given are the experimental ones with PBE-TS optimized ones in parentheses.

	succinic acid β	coronene γ	desloratadine I
**CSD REFCODE**	SUCACB03	CORONE03	GEHXEX
**Temp Exptal /K**	77	100	100
**Space group, Z'**	$P2_1/c$	$P2_1/n$	$P2_1$
**Z' (Z)**	Z'=0.5 (Z=2)	Z'=0.5 (Z=2)	Z'=1 (Z=2)
**a (Å)**	5.464 (5.4870)	10.0086 (9.9599)	6.9336 (6.8932)
**b (Å)**	8.766 (8.7162)	4.6651 (4.5891)	11.998 (12.0800)
**c (Å)**	5.004 (5.0909)	15.5437 (15.4387)	9.4691 (9.2954)
**β (°)**	93.29 (91.66)	106.576 (106.77)	107.365 (107.05)
***RMSD$_{20}$ /Å**	0.078	0.091	0.108

*RMSD$_{20}$ is the minimum root mean square difference in the non-hydrogenic atomic positions in a 20-molecule co-ordination cluster between the PBE-TS lattice energy optimised structure used to calculate the phonons and the experimental structure.

Full periodic PBE-TS optimization gave a consistent model for the molecules within the static lattice at 0 K. The optimized crystal structures are in very good agreement with the experimental low temperature structure determinations.

C. Phonon calculations and thermodynamic corrections for the crystals

PBE-TS harmonic phonon calculations were performed on the PBE-TS optimized structures, using either linear response or finite differencing algorithm with a supercell selected to ensure no imaginary frequency across the phonon Brillouin zone. A tighter SCF energy convergence tolerance (10^{-12} eV) was used to ensure properly converged numerical derivatives during phonon calculations. Details of phonon calculations on desloratadine can be found in reference,[10] and the same method was used for coronene. For the β form of succinic acid, a slightly different approach to that in reference[2] was used in its phonon calculation to remove a small but persistent region of imaginary frequencies, using norm-conserving pseudopotentials and linear response theory in CASTEP. This leads to a phonon dispersion free from any imaginary frequencies. The details of the calculations are summarised in Table S2.

Table S2 Calculation parameters and methods used in the PBE-TS optimizations and phonon calculations of crystal structures of succinic acid β, desloratadine I and coronene γ forms.

Molecule	CASTEP ver.	Pseudopotential	Planewave cut-off energy (eV)	k-point grid spacing (Å^{-1})	Phonon method	Phonon Supercells
**Succinic acid β**	18.1	On-the-fly norm-conserving	1200	0.10	Linear response	4x2x4
**Desloratadine I**	17.2	On-the-fly ultrasoft	1100	0.05	Finite differencing	2x1x1
**Coronene γ**	17.2	On-the-fly ultrasoft	1000	0.05	Finite differencing	2x4x1

Once a phonon calculation was completed, the phonon Brillouin zone was sampled with a finer nuclear Brillouin point (Γ-point) spacing and the resultant phonon density of states integrated to obtain the thermodynamic corrections for the crystal as listed in Table S3.

Table S3 Harmonic thermodynamic corrections for the succinic acid β, coronene γ and desloratadine I forms calculated from PBE-TS harmonic phonons.

In kJ/mol (T = 298.15 K)	**succinic acid β**	**coronene γ**	**desloratadine I**
Crystal ZPE E^{s}_{ZPE} (PBE-TS)	275.25	722.72	875.48
Crystal U^{s}_{corr} (PBE-TS)	297.90	764.87	926.90
Crystal A^{s}_{corr} (PBE-TS)	253.26	685.63	826.57

D. Lattice energies

As shown in Section I.A, the lattice energy contribution dominates the heat of sublimation, so its calculation deserves careful consideration. We have considered two different approaches to calculating the lattice energy:

II.D.1 using periodic DFT-D, which is widely used for relative lattice energies in crystal structure prediction,[23] but where the absolute lattice energies calculated with PBE-TS, the method used for geometry optimization of the crystals II.B, can be over-binding.[24,25,26] The effect of improving the dispersion corrections by taking into account the multi-body interactions such as MBD*,[27] which is often an improvement,[25,26] is the only currently affordable variation we investigated. This we categorise as a Ψ_{crys}-based method.[19]

II.D.2 using a model intermolecular pair potential, which has been parametrised by fitting to crystal structures and some heats of sublimation, in conjunction with an electrostatic model derived from the DFT charge distribution of the molecule, i.e. a Ψ_{mol}-based method.[19] The conformational energy penalty for shifting the molecule away from its lowest energy conformation is also obtained from the molecular electronic structure calculation. In the case of desloratadine, this corresponds to the difference between the SAA and AAA conformations.

It is possible to use better quality functionals and basis sets for the molecular electronic structure calculations (II.D.2) than for periodic ones (II.D.1). Hence for both the Ψ_{mol} approach to the lattice energy and the gas phase molecular calculations (II.D), the hybrid PBE0 functional is considered as well as the PBE functional used for the phonon calculations (II.B) and two sizes of basis set. The use of a polarisable continuum model (PCM) to approximate the change in the molecular charge distribution upon going into the crystalline phase was also considered. The results for the lattice energy (Table S5) and gas-phase properties (Table S6) are combined in Table S7 and used to develop our protocols (II.A). The data relating to our best sublimation protocols are shaded in yellow.

1. Periodic DFT-D lattice energies

The total PBE-TS electronic energies of the crystals were also obtained in the process of optimizing the three crystal structures in Section II.B. To test the effect of different dispersion corrections on the absolute lattice energies, single-point calculations at the optimized structures using the MBD* dispersion correction[27] were also performed, using the same plane wave cut-off energy and k-point grid spacing.

To calculate the periodic DFT-D lattice energy, the energies of an isolated molecule-in-a-box were subtracted from the total energy of each polymorph. The starting point for each optimisation of succinic acid and coronene was the optimized gas phase global minimum obtained during the DMACRYS calculations in Section II.D.2. For desloratadine it was the

minimised AAA conformer, which means that the periodic DFT-D sublimation free energy (but not the version using model potentials) here is computed between AAA conformers in both crystal and gas. In each case, the molecule was placed in a cubic box and then had its atomic positions optimized with a fixed unit cell. It is important to choose a large enough cubic box to minimise the interaction of the molecule with its periodic copies. To obtain an optimal box size, the molecule was put in a large cubic box, with the side length of the cubic box increased through a series of single-point PBE-TS energy calculations to make sure the energy change is small enough (< 0.2 kJ/mol for desloratadine, < 0.1 kJ/mol for the others). This ends up with the following table for the optimal side length of the box for each molecule:

Molecule-in-a-box	Succinic acid	Coronene	Desloratadine
Side-length (Å) of the cubic box	15	20	25

Using the selected cubic box, the PBE-TS molecule-in-a-box optimization converges in a few steps. The optimized molecule-in-a-box structure was also used in the PBE-MBD* single-point energy calculation for the PBE-MBD* lattice energy. Each molecule-in-a-box calculation was performed using the same plane wave cut-off energies and other convergence criteria used for the corresponding crystal structure Table S2 .

2. Lattice energies using model intermolecular potentials and the Ψ_{mol} approach

The Ψ_{mol} approach partitions the lattice energy into intermolecular energy of separating the molecules in the crystal to infinite distance, U_{inter} and the conformational energy change associated with this process, ΔE_{intra}. The dispersion-repulsion contributions to U_{inter} were calculated with an isotropic atom-atom e*xp*-6 force field, using the FIT parameters[19] which had been empirically fitted to experimental crystal structures and some heats of sublimation. The electrostatic contribution to U_{inter} was calculated from the distributed multipole representation of the DFT molecular charge distribution. Within a crystal the molecules are polarized by the crystal environment, which can be approximated by calculating the distributed multipoles within a polarisable continuum model (PCM), using the IEFPCM default in Gaussian09[13] and a dielectric constant of 3.0, typical for organic crystals.[23]

The conformational energy ΔE_{intra} is calculated using Gaussian09[13] as the energy difference between the optimized global minimum of the molecule and the molecule in its crystal conformation:

$$E_{latt} = U_{inter} + \Delta E_{intra} = U_{inter} + E_{mol\_in\_cryst} - E_{mol\_min}. \quad (13)$$

For each molecule, the molecular structure in the crystal conformation $E_{mol\_in\_cryst}$ is extracted from the PBE-TS optimised crystal structure (Table S1). Distributed multipoles were calculated with GDMA[28] from a PBE/6-311++G(2d,p) or PBE0/6-31G(d,p) charge density, calculated using Gaussian09 with or without a PCM model (ε = 3.0). This also provides the energy of the molecule in its crystal conformation ($E_{mol\_in\_cryst}$) in the specific model.

DMACRYS[19] was then used to calculate the intermolecular energies U_{inter}, as the final energy in the re-optimization of the PBE-TS optimized crystal structures (Table S1) keeping the molecule rigid for each functional/basis set/PCM combination, using distributed multipoles calculated from the corresponding charge densities and the FIT potential (Table S4). To obtain the conformational energy (ΔE_{intra}) relative to the global minimum, the extracted molecular conformation was optimized with PBE0/6-31G(d,p) or PBE/6-311++G(2d,p), again with and without the PCM model. In the cases of succinic acid and desloratadine, starting from the

extracted conformations led to the closest local stationary points on the potential energy surfaces, and so the starting structure was changed so the optimization could locate the global minimum, with an energy of E_{mol_min}. ΔE_{intra} was calculated and combined with U_{inter} to obtain the DMACRYS lattice energy, see equation (13) and Table S5.

The re-optimization of cell parameters led to only small changes, as shown in Table S4. Although all the Ψ_{mol} models reproduced the PBE-TS structures fairly well, the PCM-reoptimized structures with the PBE/6-311++G(2d,p)/PCM electrostatic model are slightly closer to the experimental and PBE-TS optimized ones. The marginal improvement in the reproduction of the crystal by the Ψ_{mol} models may be because the FIT potential was derived by fitting to experimental crystal structures and so includes some thermal expansion and zero-point energy effects in the lattice energy.

Table S4 Cell parameters after rigid-molecule optimization using DMACRYS with various functional/basis set/PCM combinations for the Ψ_{mol} calculation used for the electrostatic model. The structural change is measured by $RMSD_{20}$. the minimum root mean square difference in the non-hydrogenic atomic positions in a 20 molecule co-ordination cluster.

	**succinic acid β**	**coronene γ**	**desloratadine I**
**PBE/6-311++G(2d,p)/PCM**			
**a (Å)**	5.3787	10.0919	6.9510
**b (Å)**	8.8596	4.5780	12.2172
**c (Å)**	5.0933	16.5289	9.5966
**β (°)**	94.29	110.20	106.94
**$RMSD_{20}$ /Å (vs. PBE-TS)**	0.151	0.285	0.181
**$RMSD_{20}$ /Å (vs. Expt.)**	0.131	0.253	0.161
**PBE/6-311++G(2d,p)**			
**a (Å)**	5.3804	10.1193	6.9570
**b (Å)**	8.8939	4.5176	12.2158
**c (Å)**	5.1105	16.7342	9.6204
**β (°)**	94.65	110.43	106.94
**$RMSD_{20}$ /Å (vs. PBE-TS)**	0.177	0.355	0.193
**$RMSD_{20}$ /Å (vs. Expt.)**	0.149	0.334	0.168
**PBE0/6-31G(d,p)/PCM**			
**a (Å)**	5.4091	10.0824	6.9561
**b (Å)**	8.8609	4.6057	12.2248
**c (Å)**	5.1147	16.4488	9.6006
**β (°)**	94.40	110.17	107.01
**$RMSD_{20}$ /Å (vs. PBE-TS)**	0.173	0.259	0.183
**$RMSD_{20}$ /Å (vs. Expt.)**	0.140	0.219	0.164
**PBE0/6-31G(d,p)**			
**a (Å)**	5.4128	10.1117	6.9620
**b (Å)**	8.8784	4.5338	12.2237
**c (Å)**	5.1272	16.6878	9.6188
**β (°)**	94.62	110.41	107.01
**$RMSD_{20}$ /Å (vs. PBE-TS)**	0.185	0.336	0.192
**$RMSD_{20}$ /Å (vs. Expt.)**	0.152	0.313	0.169

3. Choice of lattice energy model

Table S5 Lattice energies of the three crystal structures calculated in various approaches. The yellow shaded row shows the results calculated with the protocol in Section II.C.

**Lattice Energies in kJ/mol**	**succinic acid β**	**coronene γ**	**desloratadine I**
Ψ_{mol}-based methods			
DMACRYS PBE/6-311++G(2d,p)/PCM	-125.89	-155.61	-144.40
DMACRYS PBE/6-311++G(2d,p)	-110.87	-156.08	-138.44
DMACRYS PBE0/6-31G(d,p)/PCM	-103.22	-155.36	-141.86
DMACRYS PBE0/6-31G(d,p)	-92.35	-155.94	-136.37
Ψ_{crys}-based methods			
CASTEP PBE-TS	-148.43	-205.09	-202.37 **
CASTEP PBE-MBD*/PBE-TS	-139.42	-161.13	-173.30 **

** the AAA conformation was used in the molecular calculation.

The periodic PBE-TS lattice energies from the CASTEP Ψ_{crys} calculations in Table S5 are considerably larger than those calculated with Ψ_{mol}-based methods and give a large error when used to estimate heats of sublimation or solubilities. The MBD* dispersion correction is a significant improvement, emphasising the importance of the dispersion energy, but still the absolute lattice energies with the PBE functional are unrealistic, as has been found in other studies. [29,24,25,26]

The Ψ_{mol}-based DMACRYS lattice energies (Table S5) show a much larger variation with functional, basis set, or use of a PCM model for β succinic acid than for coronene or desloratadine. Compared to the reference experimental lattice energy for the β form of succinic acid in the revised X23 set[26] (-130.1 kJ/mol), DMACRYS lattice energies were all underestimated, with the best DMACRYS lattice energy given by the PBE/6-311++G(2d,p)/PCM combination. For this reason, PBE/6-311++G(2d,p)/PCM multipoles were chosen for the sublimation free energy contribution to our solubility predictions, and sublimation data obtained with this method are shaded yellow in the tables. The Ψ_{mol} lattice energy for desloratadine and succinic acid includes the conformational energy penalty that arises because the molecule changes conformation between the gas-phase and the crystal phase.

E. Thermodynamic corrections for gas-phase molecules

For each molecule optimized in Section III.C.1, the molecular vibrations were calculated with Gaussian09 using the same functional/basis set/PCM combination. Thermal analysis by Gaussian09 then provided the E^g_{ZPE}, H^g_{corr}, A^g_{corr} for the global minimum, which are included in solubility predictions. The "Tight" convergence criteria within Gaussian09 were used along with an ultrafine integration grid from two-electron integrals and their derivatives. Although this has little influence on the total energy, it ensures more accurate calculations of low-frequency vibrational modes and it affected the thermochemistry calculations, particularly for coronene.

As shown in Table S6, there is a significant difference between the thermodynamic corrections calculated using the PBE0/6-31G(d,p) and PBE/6-311++G(2d,p) combinations, since the molecular frequencies calculated were systematically different. Table S6 shows a much smaller difference between the thermodynamic corrections with or without the PCM model. The largest

such difference is ~ 2kJ/mol, enough to be significant for $\log S_0$, for A^g_{corr} of coronene at the PBE/6-311++G(2d,p) level.

Table S6 Calculated thermodynamic data using different functional/basis set/PCM combination for the three molecules.

Molecular thermal corrections In kJ/mol	succinic acid (gauche)	coronene	desloratadine (SAA)
E^g_{ZPE}			
DMACRYS PBE/6-311++G(2d,p)/PCM	268.55	711.53	865.26
DMACRYS PBE/6-311++G(2d,p)	269.20	711.18	865.57
DMACRYS PBE0/6-31G(d,p)/PCM	281.76	741.05	901.98
DMACRYS PBE0/6-31G(d,p)	282.61	740.92	902.27
H^g_{corr}			
DMACRYS PBE/6-311++G(2d,p)/PCM	293.05	752.77	916.22
DMACRYS PBE/6-311++G(2d,p)	293.64	752.44	916.44
DMACRYS PBE0/6-31G(d,p)/PCM	305.56	780.47	950.99
DMACRYS PBE0/6-31G(d,p)	306.28	780.36	951.22
A^g_{corr}			
DMACRYS PBE/6-311++G(2d,p)/PCM	176.43	606.59	739.46
DMACRYS PBE/6-311++G(2d,p)	177.28	604.57	739.91
DMACRYS PBE0/6-31G(d,p)/PCM	190.46	634.26	778.19
DMACRYS PBE0/6-31G(d,p)	191.98	634.11	778.61

Comparing the molecular frequencies in Table S6 to the crystalline thermodynamic corrections calculated from periodic PBE-TS phonon calculations in Table S3, it becomes clear that the systematic differences between the PBE0 and PBE functionals are a potential source of error for the calculation of solubility. The PBE0/6-31G(d,p) calculations should provide better molecular vibrational frequencies and hence better thermodynamics for the gas phase. However, for optimising the cancellation of errors in the thermodynamic cycle for solubility calculations, the gas phase properties were calculated with the same functional (PBE) and basis set (6-311++G(2d,p)) using PCM.

III. Results

A. Thermodynamics of sublimation

Combining DMACRYS and periodic DFT-D lattice energies (Table S5) and thermodynamic corrections from periodic PBE-TS (Table S3) and the corresponding molecular models (Table S6) gives the thermodynamic properties of sublimation (Table S7). Compared with the experimental values for β succinic acid and γ coronene, the best results are given by using the PBE/6-311++G(2d,p)/PCM Ψ_{mol} lattice energies. The periodic DFT-D lattice energies were

always overestimated, with those using the MBD* dispersion correction showing a marked improvement over those using the pairwise additive TS dispersion correction.

The use of a PCM model significantly improves the calculated heat of sublimation for β succinic acid, slightly changes that for desloratadine form I, but does not affect the coronene results. This reflects the relative importance of the electrostatic contribution to U_{inter} , which is most significant in succinic acid, smaller in desloratadine, which has fewer hydrogen bonds relative to the size of the molecule, and small in coronene. With a PCM model, U_{inter} between succinic acid molecules increases by more than 12 kJ/mol, and the PCM model stabilizes the elongated O-H bonds of succinic acid in the crystal, reducing ΔE_{intra} by 3 kJ/mol. Together this shifts the ΔH_{sub} significantly closer to the experimental value. It appears that using the PCM model for all phases helps the cancellation of errors due to the change in O-H bond lengths between phases.

Table S7 Thermodynamics of sublimation of β succinic acid, γ coronene and desloratadine form I, calculated using thermodynamic corrections from PBE-TS phonons and a variety of methods for the other components. All thermodynamic quantities are given in kJ/mol and correspond to T = 298.15 K.

Lattice energy E_{latt}	ΔH°_{sub}	ΔA°_{sub}	ΔG°_{sub}	$T\Delta S^{\circ}_{sub}$	$\Delta H^{\circ}_{sub} + E_{latt}$ **
**succinic acid β (expt. ΔH_{sub} = 123.2)**					
PBE/6-311++G(2d,p)/PCM	121.04	49.06	51.54	69.50	-4.85
PBE/6-311++G(2d,p)	106.61	34.89	37.37	69.24	-4.26
PBE0/6-31G(d,p)/PCM	110.88	40.42	42.90	67.98	7.66
PBE0/6-31G(d,p)	100.73	31.07	33.55	67.18	8.38
+PBE-TS	143.58	71.60	74.08	69.50	-4.85
+PBE-MBD*	134.57	62.59	65.07	69.50	-4.85
**coronene γ (expt. ΔH_{sub} = 148.2)**					
PBE/6-311++G(2d,p)/PCM	143.51	76.57	79.05	64.46	-12.1
PBE/6-311++G(2d,p)	143.65	75.02	77.50	66.15	-12.43
PBE0/6-31G(d,p)/PCM	170.96	103.99	106.47	64.49	15.6
PBE0/6-31G(d,p)	171.43	104.42	106.90	64.53	15.49
+PBE-TS	192.99	126.05	128.53	64.46	-12.1
+PBE-MBD*	149.03	82.09	84.57	64.46	-12.1
**desloratadine I**					
PBE/6-311++G(2d,p)/PCM	133.72	57.29	59.77	73.95	-10.68
PBE/6-311++G(2d,p)	127.98	51.78	54.26	73.72	-10.46
PBE0/6-31G(d,p)/PCM	165.95	93.48	95.96	69.99	24.09
PBE0/6-31G(d,p)/PCM	160.69	88.41	90.89	69.80	24.32
+PBE-TS	191.69	115.26	117.74	73.95	-10.68
+PBE-MBD*	162.62	86.19	88.67	73.95	-10.68

+ Gas-phase thermodynamic corrections from PBE/6-311++G(2d,p)/PCM calculations were used with periodic DFT-D lattice energies. ** This quantity is often approximated as -2RT=-4.96 kJ/mol.

Solvation Free Energy Calculations

I. Methods

A. Additional Information about the Clustering Method used in the Calculation of SFE1 and SFE3.

In calculating SFE1 and SFE3, to avoid double counting identical states in Boltzmann-weighted averages, the low-energy structures produced by the conformational search algorithm were clustered to remove duplicates. Using succinic acid as an example, the force-field based conformational search (described in the "Computational Methods" section of the main article) produced 432 conformers. These structures were each re-optimised at the appropriate level of DFT theory (e.g. PBE, PBE0, or PBE0-DH with the 6-311++G(2d,p) basis set) in the appropriate phase (e.g. gas or water modelled using SMD). The resulting re-optimised conformers were then sorted into groups with identical calculated energy. This typically gave between 10 and 20 groups, depending on which DFT method was used and which phase was being modelled, e.g. there were 14 groups for succinic acid modelled using PBE/6-311++G(2d,p) in the gas-phase. Within each group, a distance matrix was then calculated by aligning each pair of conformers using the "obfit" command in OpenBabel and recording the root-mean-square-deviation (RMSD) in the atomic coordinates. Only one of each pair of conformers with a RMSD < 0.1 Å was retained in the Boltzmann-weighted averages. This typically removed all but one conformer from each group. Sensitivity testing suggested that modifying the RMSD cutoff between 0.05 and 0.5Å had a negligible effect on the results.

B. Comment on Thermal Corrections to the Solvation Free Energy

The non-polar terms in the SMD model for water were initially parameterised and validated against experimental data for organic molecules.[30] Since most of those molecules were small with few rotatable bonds, the authors of that work were able to make several simplifications to the quantum chemical calculations without loss of accuracy, namely: (i) using the same conformer in gas and solution phase; (ii) not calculating the thermal corrections to the *ab initio* or DFT energy. For larger non-rigid solutes, it is usually preferable to re-optimise the molecular structure in gas and solution-phase separately, to account for the conformational rearrangement energy (this is the default approach in most quantum chemical software packages, including Gaussian16, and this was done here). However, there has been some disagreement in the literature about whether thermal corrections should be calculated,[31,32] with most of the discussion focusing on whether they are implicitly included in the parameterised non-polar terms or not. From a practical point-of-view, it influences the computational expense since a frequency calculation in each phase is required to obtain contributions from the vibrational partition function. Comparing these two approaches to experimental data for 25 molecules from the Minnesota Solvation Database suggests that the extra computational expense in obtaining the thermal corrections is not justified. Since we did not know whether that conclusion would hold for the solubility dataset, however, results with and without thermal corrections were evaluated, and are presented in Table S8.

II. Results

A. Minnesota Solvation Database

A dataset of 130 small drug-like molecules were obtained from the Minnesota Solvation Database and used for benchmarking hydration free energies at the PBE/6-311++G(2d,p)/SMD and PBE0-DH/6-311++G(2d,p)/SMD levels of theory.

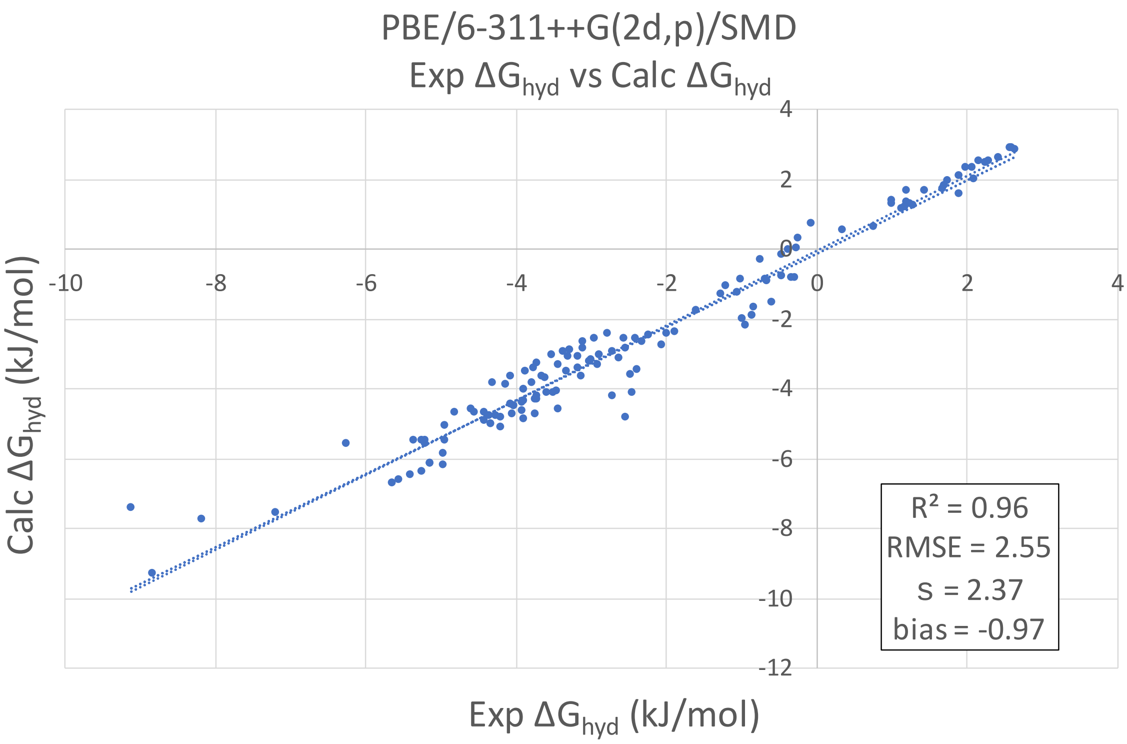

Figure S2 Correlation between experimental and calculated hydration free energy for 130 molecules from the Minnesota Solvation Database. Calculated values were obtained at the PBE/6-311++G(2d,p)/SMD level of theory.

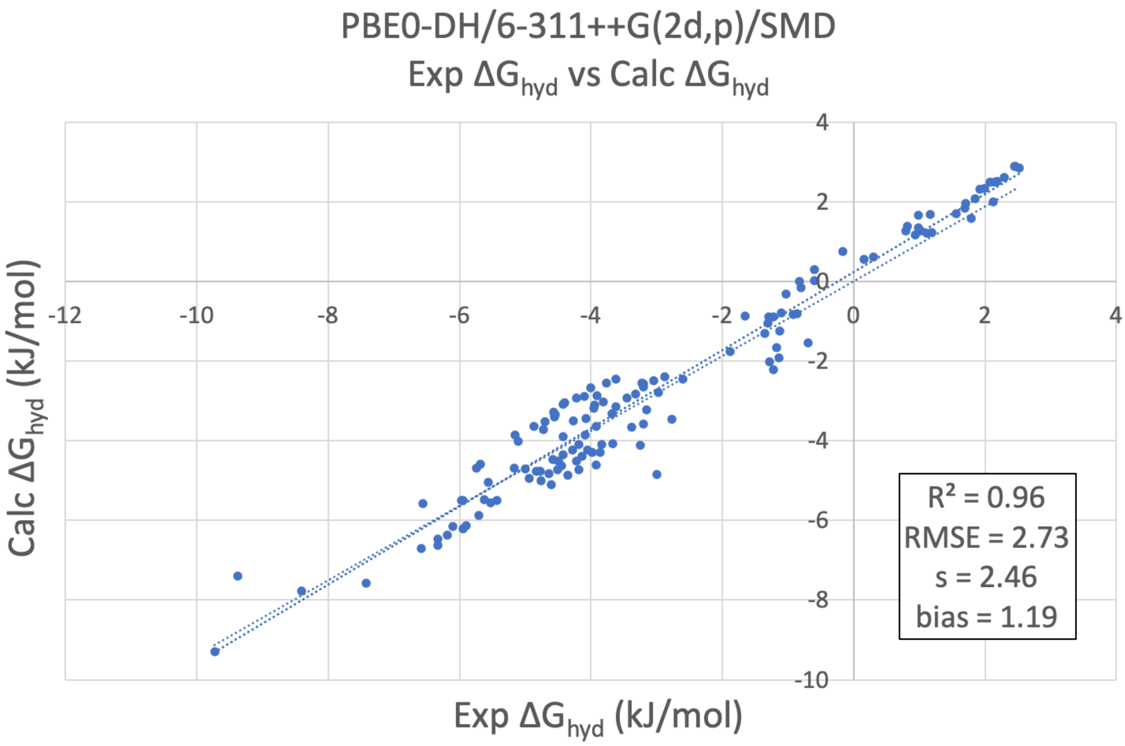

Figure S3 Correlation between experimental and calculated hydration free energy for 130 molecules from the Minnesota Solvation Database. Calculated values were obtained at the PBE0-DH/6-311++G(2d,p)/SMD level of theory.

B. Succinic Acid, Desloratadine, and Coronene

Predicted hydration free energies for all three compounds are shown in Table S8. Numbers resulting from our preferred protocols are shaded in green. The experimental hydration free energy for coronene is obtained by back-calculation from the known solubility and sublimation enthalpy[8,9] under the assumptions detailed in the footnote to Table 2 in the accompanying article.

Table S8 Summary of experimental[5] and calculated hydration free energies. All energy values are given in kJ/mol. LogS_0 was predicted from three different treatments of conformations: SFE1, SFE2, and SFE3, as defined in the accompanying article. Calculated hydration free energies are given for the PBE/6-311++G(2d,p)/SMD, PBE0/6-311++G(2d,p)/SMD and PBE0DH/6-311++G(2d,p)/SMD levels of theory. Results used to compute logS_0 in the paper are shaded green.

Molecule	ΔG_{hyd}(exp) [kJ/mol]	SFEn	Method	ΔG_{hyd}(SCF) [kJ/mol]	ΔG_{hyd} (SCF+ZPE) [kJ/mol]	ΔG_{hyd} (SCF+Therm) [kJ/mol]
Coronene	-38.40	SFE2	PBE	-18.68	-17.90	-17.85
			PBE0	-23.01	-22.30	-22.26
			PBE0DH	-26.32	-25.61	-25.57
Desloratadine		SFE1	PBE	-44.91	-44.64	-44.92
			PBE0	-47.88	-47.86	-48.01
			PBE0DH	-49.17	-49.05	-48.88
		SFE2	PBE	-45.11	-44.84	-45.13
			PBE0	-48.08	-48.05	-48.22
			PBE0DH	-50.38	-50.35	-50.52
		SFE3	PBE	-44.74	-44.12	-44.27
			PBE0	-46.57	-46.32	-46.44
			PBE0DH	-51.11	-51.00	-51.02
Succinic acid	-61.08	SFE1	PBE	-49.20	-51.65	-54.18
			PBE0	-52.65	-55.39	-59.97
			PBE0DH	-56.13	-56.40	-59.95
		SFE2	PBE	-49.33	-51.77	-53.52
			PBE0	-52.78	-55.51	-57.41
			PBE0DH	-56.23	-56.49	-58.65
		SFE3	PBE	-49.27	-51.72	-51.52
			PBE0	-52.73	-55.47	-54.16
			PBE0DH	-56.20	-56.48	-55.89

Solubility Prediction from Sublimation and Hydration Free Energies

Calculated values of ΔG°_{sub} and ΔG^{*}_{hydr} are combined to compute $\log S_0$, for each combination of sublimation and hydration models, using equation (12). The results are shown in Table S9.

Table S9 Calculated $\log S_0$ values for coronene, desloratadine, and succinic acid obtained from different combinations of sublimation and hydration models, computed using equation (12). SFE 1-3 are defined as for Table S8. $\log S_0$ values were calculated using hydration free energies computed from DFT energy only (A), DFT energy plus zero-point-energy only (B), or DFT energy plus all thermal corrections (C).

Molecule		Hydration Method	Sublimation Method	log S (A)	log S (B)	log S (C)	Error log S (A)	Error log S (B)	Error log S (C)
Coronene $\log S_{exp}$=-9.33	SFE2	PBE 6-311++G(2d,p) SMD	PBE/6-311++G(2d,p)/PCM	-11.96	-12.10	-12.11	2.63	2.77	2.78
			PBE/6-311++G(2d,p)	-11.69	-11.83	-11.84	2.36	2.50	2.51
			PBE0/6-31G(d,p)/PCM	-16.77	-16.91	-16.91	7.44	7.58	7.58
			PBE0/6-31G(d,p)	-16.84	-16.98	-16.99	7.51	7.65	7.66
			+PBE-TS	-20.63	-20.77	-20.78	11.30	11.44	11.45
			+PBE-MBD	-12.93	-13.07	-13.08	3.60	3.74	3.75
		PBE0 6-311++G(2d,p) SMD	PBE/6-311++G(2d,p)/PCM	-11.21	-11.33	-11.34	1.88	2.00	2.01
			PBE/6-311++G(2d,p)	-10.93	-11.06	-11.07	1.60	1.73	1.74
			PBE0/6-31G(d,p)/PCM	-16.01	-16.13	-16.14	6.68	6.80	6.81
			PBE0/6-31G(d,p)	-16.09	-16.21	-16.22	6.76	6.88	6.89
			+PBE-TS	-19.87	-20.00	-20.01	10.54	10.67	10.68
			+PBE-MBD	-12.17	-12.30	-12.30	2.84	2.97	2.97
		PBE0-DH 6-311++G(2d,p) SMD	PBE/6-311++G(2d,p)/PCM	-10.63	-10.75	-10.76	1.30	1.42	1.43
			PBE/6-311++G(2d,p)	-10.35	-10.48	-10.49	1.02	1.15	1.16
			PBE0/6-31G(d,p)/PCM	-15.43	-15.55	-15.56	6.10	6.22	6.23
			PBE0/6-31G(d,p)	-15.51	-15.63	-15.64	6.18	6.30	6.31
			+PBE-TS	-19.29	-19.42	-19.43	9.96	10.09	10.10
			+PBE-MBD	-11.59	-11.72	-11.72	2.26	2.39	2.39
Desloratadine $\log S_{exp}$=-3.42	SFE1	PBE 6-311++G(2d,p) SMD	PBE/6-311++G(2d,p)/PCM	-3.99	-4.04	-3.99	0.57	0.62	0.57
			PBE/6-311++G(2d,p)	-3.03	-3.07	-3.02	-0.39	-0.35	-0.40
			PBE0/6-31G(d,p)/PCM	-10.33	-10.38	-10.33	6.91	6.96	6.91
			PBE0/6-31G(d,p)/PCM	-9.44	-9.49	-9.44	6.02	6.07	6.02
			+PBE-TS	-14.15	-14.20	-14.15	10.73	10.78	10.73
			+PBE-MBD	-9.05	-9.10	-9.05	5.63	5.68	5.63
		PBE0 6-311++G(2d,p) SMD	PBE/6-311++G(2d,p)/PCM	-3.47	-3.48	-3.45	0.05	0.06	0.03
			PBE/6-311++G(2d,p)	-2.51	-2.51	-2.48	-0.91	-0.91	-0.94
			PBE0/6-31G(d,p)/PCM	-9.81	-9.82	-9.79	6.39	6.40	6.37
			PBE0/6-31G(d,p)/PCM	-8.92	-8.93	-8.90	5.50	5.51	5.48

Molecule	Hydration Method	Sublimation Method	log S (A)	log S (B)	log S (C)	Error log S (A)	Error log S (B)	Error log S (C)
		+PBE-TS	-13.63	-13.63	-13.60	10.21	10.21	10.18
		+PBE-MBD	-8.53	-8.54	-8.51	5.11	5.12	5.09
	PBE0-DH 6-311++G(2d,p) SMD	PBE/6-311++G(2d,p)/PCM	-3.25	-3.27	-3.30	-0.17	-0.15	-0.12
		PBE/6-311++G(2d,p)	-2.28	-2.30	-2.33	-1.14	-1.12	-1.09
		PBE0/6-31G(d,p)/PCM	-9.59	-9.61	-9.64	6.17	6.19	6.22
		PBE0/6-31G(d,p)/PCM	-8.70	-8.72	-8.75	5.28	5.30	5.33
		+PBE-TS	-13.40	-13.42	-13.45	9.98	10.00	10.03
		+PBE-MBD	-8.31	-8.33	-8.36	4.89	4.91	4.94
SFE2	PBE 6-311++G(2d,p) SMD	PBE/6-311++G(2d,p)/PCM	-3.96	-4.00	-3.95	0.54	0.58	0.53
		PBE/6-311++G(2d,p)	-2.99	-3.04	-2.99	-0.43	-0.38	-0.43
		PBE0/6-31G(d,p)/PCM	-10.30	-10.34	-10.29	6.88	6.92	6.87
		PBE0/6-31G(d,p)/PCM	-9.41	-9.46	-9.41	5.99	6.04	5.99
		+PBE-TS	-14.11	-14.16	-14.11	10.69	10.74	10.69
		+PBE-MBD	-9.02	-9.07	-9.02	5.60	5.65	5.60
	PBE0 6-311++G(2d,p) SMD	PBE/6-311++G(2d,p)/PCM	-3.44	-3.44	-3.41	0.02	0.02	-0.01
		PBE/6-311++G(2d,p)	-2.47	-2.48	-2.45	-0.95	-0.94	-0.97
		PBE0/6-31G(d,p)/PCM	-9.78	-9.78	-9.75	6.36	6.36	6.33
		PBE0/6-31G(d,p)/PCM	-8.89	-8.89	-8.86	5.47	5.47	5.44
		+PBE-TS	-13.59	-13.60	-13.57	10.17	10.18	10.15
		+PBE-MBD	-8.50	-8.50	-8.48	5.08	5.08	5.06
	PBE0-DH 6-311++G(2d,p) SMD	PBE/6-311++G(2d,p)/PCM	-3.03	-3.04	-3.01	-0.39	-0.38	-0.41
		PBE/6-311++G(2d,p)	-2.07	-2.07	-2.04	-1.35	-1.35	-1.38
		PBE0/6-31G(d,p)/PCM	-9.37	-9.38	-9.35	5.95	5.96	5.93
		PBE0/6-31G(d,p)/PCM	-8.49	-8.49	-8.46	5.07	5.07	5.04
		+PBE-TS	-13.19	-13.19	-13.16	9.77	9.77	9.74
		+PBE-MBD	-8.10	-8.10	-8.07	4.68	4.68	4.65
SFE3	PBE 6-311++G(2d,p) SMD	PBE/6-311++G(2d,p)/PCM	-4.02	-4.13	-4.10	0.60	0.71	0.68
		PBE/6-311++G(2d,p)	-3.06	-3.17	-3.14	-0.36	-0.25	-0.28
		PBE0/6-31G(d,p)/PCM	-10.36	-10.47	-10.44	6.94	7.05	7.02
		PBE0/6-31G(d,p)/PCM	-9.47	-9.58	-9.56	6.05	6.16	6.14
		+PBE-TS	-14.18	-14.29	-14.26	10.76	10.87	10.84
		+PBE-MBD	-9.08	-9.19	-9.17	5.66	5.77	5.75
	PBE0 6-311++G(2d,p) SMD	PBE/6-311++G(2d,p)/PCM	-3.70	-3.74	-3.72	0.28	0.32	0.30
		PBE/6-311++G(2d,p)	-2.74	-2.78	-2.76	-0.68	-0.64	-0.66
		PBE0/6-31G(d,p)/PCM	-10.04	-10.09	-10.06	6.62	6.67	6.64
		PBE0/6-31G(d,p)/PCM	-9.15	-9.20	-9.18	5.73	5.78	5.76
		+PBE-TS	-13.86	-13.90	-13.88	10.44	10.48	10.46

Molecule		Hydration Method	Sublimation Method	log S (A)	log S (B)	log S (C)	Error log S (A)	Error log S (B)	Error log S (C)
			+PBE-MBD	-8.76	-8.81	-8.79	5.34	5.39	5.37
		PBE0-DH 6-311++G(2d,p) SMD	PBE/6-311++G(2d,p)/PCM	-2.91	-2.92	-2.92	-0.51	-0.50	-0.50
			PBE/6-311++G(2d,p)	-1.94	-1.96	-1.96	-1.48	-1.46	-1.46
			PBE0/6-31G(d,p)/PCM	-9.25	-9.27	-9.26	5.83	5.85	5.84
			PBE0/6-31G(d,p)/PCM	-8.36	-8.38	-8.37	4.94	4.96	4.95
			+PBE-TS	-13.06	-13.08	-13.08	9.64	9.66	9.66
			+PBE-MBD	-7.97	-7.99	-7.98	4.55	4.57	4.56
Succinic acid logS$_{exp}$=-0.22	SFE1	PBE 6-311++G(2d,p) SMD	PBE/6-311++G(2d,p)/PCM	-1.80	-1.37	-0.93	1.58	1.15	0.71
			PBE/6-311++G(2d,p)	0.68	1.11	1.56	-0.90	-1.33	-1.78
			PBE0/6-31G(d,p)/PCM	-0.28	0.14	0.59	0.06	-0.36	-0.81
			PBE0/6-31G(d,p)	1.35	1.78	2.23	-1.57	-2.00	-2.45
			+PBE-TS	-5.75	-5.32	-4.87	5.53	5.10	4.65
			+PBE-MBD	-4.17	-3.74	-3.30	3.95	3.52	3.08
		PBE0 6-311++G(2d,p) SMD	PBE/6-311++G(2d,p)/PCM	-1.19	-0.71	0.09	0.97	0.49	-0.31
			PBE/6-311++G(2d,p)	1.29	1.77	2.57	-1.51	-1.99	-2.79
			PBE0/6-31G(d,p)/PCM	0.32	0.80	1.60	-0.54	-1.02	-1.82
			PBE0/6-31G(d,p)	1.96	2.44	3.24	-2.18	-2.66	-3.46
			+PBE-TS	-5.14	-4.66	-3.86	4.92	4.44	3.64
			+PBE-MBD	-3.56	-3.08	-2.28	3.34	2.86	2.06
		PBE0-DH 6-311++G(2d,p) SMD	PBE/6-311++G(2d,p)/PCM	-0.58	-0.54	0.08	0.36	0.32	-0.30
			PBE/6-311++G(2d,p)	1.90	1.95	2.57	-2.12	-2.17	-2.79
			PBE0/6-31G(d,p)/PCM	0.93	0.98	1.60	-1.15	-1.20	-1.82
			PBE0/6-31G(d,p)	2.57	2.61	3.24	-2.79	-2.83	-3.46
			+PBE-TS	-4.53	-4.49	-3.86	4.31	4.27	3.64
			+PBE-MBD	-2.95	-2.91	-2.29	2.73	2.69	2.07
	SFE2	PBE 6-311++G(2d,p) SMD	PBE/6-311++G(2d,p)/PCM	-1.78	-1.35	-1.04	1.56	1.13	0.82
			PBE/6-311++G(2d,p)	0.71	1.13	1.44	-0.93	-1.35	-1.66
			PBE0/6-31G(d,p)/PCM	-0.26	0.17	0.47	0.04	-0.39	-0.69
			PBE0/6-31G(d,p)	1.38	1.80	2.11	-1.60	-2.02	-2.33
			+PBE-TS	-5.72	-5.30	-4.99	5.50	5.08	4.77
			+PBE-MBD	-4.15	-3.72	-3.41	3.93	3.50	3.19
		PBE0 6-311++G(2d,p) SMD	PBE/6-311++G(2d,p)/PCM	-1.17	-0.69	-0.36	0.95	0.47	0.14
			PBE/6-311++G(2d,p)	1.31	1.79	2.12	-1.53	-2.01	-2.34
			PBE0/6-31G(d,p)/PCM	0.34	0.82	1.15	-0.56	-1.04	-1.37
			PBE0/6-31G(d,p)	1.98	2.46	2.79	-2.20	-2.68	-3.01
			+PBE-TS	-5.12	-4.64	-4.31	4.90	4.42	4.09
			+PBE-MBD	-3.54	-3.06	-2.73	3.32	2.84	2.51

**Molecule**	**Hydration Method**	Sublimation Method	**log S (A)**	**log S (B)**	**log S (C)**	**Error log S (A)**	**Error log S (B)**	**Error log S (C)**
	PBE0-DH 6-311++G(2d,p) SMD	PBE/6-311++G(2d,p)/PCM	-0.57	-0.52	-0.14	0.35	0.30	-0.08
		PBE/6-311++G(2d,p)	1.92	1.96	2.34	-2.14	-2.18	-2.56
		PBE0/6-31G(d,p)/PCM	0.95	0.99	1.37	-1.17	-1.21	-1.59
		PBE0/6-31G(d,p)	2.58	2.63	3.01	-2.80	-2.85	-3.23
		+PBE-TS	-4.52	-4.47	-4.09	4.30	4.25	3.87
		+PBE-MBD	-2.94	-2.89	-2.51	2.72	2.67	2.29
SFE3	PBE 6-311++G(2d,p) SMD	PBE/6-311++G(2d,p)/PCM	-1.79	-1.36	-1.39	1.57	1.14	1.17
		PBE/6-311++G(2d,p)	0.70	1.13	1.09	-0.92	-1.35	-1.31
		PBE0/6-31G(d,p)/PCM	-0.27	0.16	0.12	0.05	-0.38	-0.34
		PBE0/6-31G(d,p)	1.37	1.79	1.76	-1.59	-2.01	-1.98
		+PBE-TS	-5.74	-5.31	-5.34	5.52	5.09	5.12
		+PBE-MBD	-4.16	-3.73	-3.76	3.94	3.51	3.54
	PBE0 6-311++G(2d,p) SMD	PBE/6-311++G(2d,p)/PCM	-1.18	-0.70	-0.93	0.96	0.48	0.71
		PBE/6-311++G(2d,p)	1.30	1.78	1.55	-1.52	-2.00	-1.77
		PBE0/6-31G(d,p)/PCM	0.33	0.81	0.58	-0.55	-1.03	-0.80
		PBE0/6-31G(d,p)	1.97	2.45	2.22	-2.19	-2.67	-2.44
		+PBE-TS	-5.13	-4.65	-4.88	4.91	4.43	4.66
		+PBE-MBD	-3.55	-3.07	-3.30	3.33	2.85	3.08
	PBE0-DH 6-311++G(2d,p) SMD	PBE/6-311++G(2d,p)/PCM	-0.57	-0.52	-0.63	0.35	0.30	0.41
		PBE/6-311++G(2d,p)	1.91	1.96	1.86	-2.13	-2.18	-2.08
		PBE0/6-31G(d,p)/PCM	0.94	0.99	0.89	-1.16	-1.21	-1.11
		PBE0/6-31G(d,p)	2.58	2.63	2.53	-2.80	-2.85	-2.75
		+PBE-TS	-4.52	-4.47	-4.58	4.30	4.25	4.36
		+PBE-MBD	-2.94	-2.89	-3.00	2.72	2.67	2.78

Table S10 Calculated logS_0 values for coronene, desloratadine, and succinic acid obtained from different combinations of sublimation and hydration models, computed using equation (12). logS_0 values were calculated using hydration free energies computed from MD/FEP energy only.

Molecule	Hydration Method	Sublimation Method	log S	Error log S
Succinic acid logS_{exp}=-0.22	GAFF/AM1-BCC, SPC/E	PBE/6-311++G(2d,p)/PCM	-0.35	0.13
		PBE/6-311++G(2d,p)	2.13	-2.35
		PBE0/6-31G(d,p)/PCM	1.16	-1.38
		PBE0/6-31G(d,p)	2.80	-3.02
		$^{+}$PBE-TS	-4.30	4.08
		$^{+}$PBE-MBD	-2.72	2.50
Coronene logS_{exp}=-9.33	GAFF/AM1-BCC, SPC/E	PBE/6-311++G(2d,p)/PCM	-8.23	-1.10
		PBE/6-311++G(2d,p)	-7.96	-1.37
		PBE0/6-31G(d,p)/PCM	-13.03	3.70
		PBE0/6-31G(d,p)	-13.11	3.78
		$^{+}$PBE-TS	-16.90	7.57
		$^{+}$PBE-MBD	-9.20	-0.13
Desloratadine logS_{exp}=-3.42	GAFF/AM1-BCC, SPC/E	PBE/6-311++G(2d,p)/PCM	-3.99	0.57
		PBE/6-311++G(2d,p)	-3.02	-0.40
		PBE0/6-31G(d,p)/PCM	-10.33	6.91
		PBE0/6-31G(d,p)	-9.44	6.02
		$^{+}$PBE-TS	-14.14	10.72
		$^{+}$PBE-MBD	-9.05	5.63

References

[1] Groom, C. R.; Bruno, I. J.; Lightfoot M. P.; Ward, S. C. The Cambridge Structural Database, *Acta Cryst*, **2016**, *B72*, 171-179.

[2] Lucaioli, P.; Nauha, E.; Gimondi, I.; Price, L. S.; Guo, R.; Iuzzolino, L.; Singh, I.; Salvalaglio, M.; Price, S. L.; Blagden, N., Serendipitous isolation of a disappearing conformational polymorph of succinic acid challenges computational polymorph prediction. *CrystEngComm* **2018,** *20* (28), 3971-3977.

[3] Forbes, G.S.; Coolidge, A.S. Relations between distribution ratio, temperature and concentration in system: water, ether, succinic acid. *J. Am. Chem. Soc.*, **1919**, *41*, 150–167.

[4] Ribeiro da Silva, M.A.; Monte, M. J.; Ribeiro, J. R. Thermodynamic study on the sublimation of succinic acid and of methyl-and dimethyl-substituted succinic and glutaric acids. *J. Chem. Thermodyn.*, **2001**, *33*, 23-31.

[5] Rees, D.C.; Wolfe, G.M. Macromolecular solvation energies derived from small molecule crystal morphology. *Prot. Sci.*, **1993**, *2*, 1882-1889.

[6] Potticary, J.; Terry, L. R.; Bell, C.; Papanikolopoulos, A. N.; Christianen, P. C. M.; Engelkamp, H.; Collins, A. M.; Fontanesi, C.; Kociok-Kohn, G.; Crampin, S.; Da Como, E.; Hall, S. R. An unforeseen polymorph of coronene by the application of magnetic fields during crystal growth. *Nat. Commun.* **2016,** *7*, 11555.
[7] Potticary, J.; Boston, R.; Vella-Zarb, L.; Few, A.; Bell, C.; Hall, S. R. Low temperature magneto-morphological characterisation of coronene and the resolution of previously observed unexplained phenomena. *Sci Rep* **2016,** *6*, 38696.
[8] Miller, M.M.; Wasik, S.P.; Huang, G.-L.; Mackay, D. Relationships between octanol–water partition coefficient and aqueous solubility. *Environ. Sci. Technol.*, **1985**, *19*, 522–529.
[9] Chickos, J. S.; Webb, P.; Nichols, G.; Kiyobayashi, T.; Cheng, P. C.; Scott, L. The enthalpy of vaporization and sublimation of corannulene, coronene, and perylene at T= 298.15 K. J. Chem. Thermodyn., 2002, 34, 1195-1206.
[10] Srirambhatla, V. K.; Guo, R.; Dawson, D. M.; Price, S. L.; Florence, A. J., Reversible, Two-Step Single-Crystal to Single-Crystal Phase Transitions between Desloratadine Forms I, II, and III. *Crys. Growth Des.* **2020,** *20*, 1800-1810.
[11] Popovic, G.; Cakar, M.; Agbaba, D. Acid–base equilibria and solubility of loratadine and desloratadine in water and micellar media. *J. Pharm. Biomed.*, **2009**, *49*, 42–47.
[12] Clark, S. J.; Segall, M. D.; Pickard, C. J.; Hasnip, P. J.; Probert, M. J.; Refson, K.; Payne, M. C., First principles methods using CASTEP. *Zeitschrift fur Kristallographie* **2005**, *220*, 567-570.
[13] Frisch, M. J.; Trucks, G. W.; Schlegel, H. B.; Scuseria, G. E.; Robb, M. A.; Cheeseman, J. R.; Scalmani, G.; Barone, V.; Mennucci, B.; Petersson, G. A.; Nakatsuji, H.; Caricato, M.; Li, X.; Hratchian, H. P.; Izmaylov, A. F.; Bloino, J.; Zheng, G.; Sonnenberg, J. L.; Hada, M.; Ehara, M.; Toyota, K.; Fukuda, R.; Hasegawa, J.; Ishida, M.; Nakajima, T.; Honda, Y.; Kitao, O.; Nakai, H.; Vreven, T.; Montgomery, J. A., Jr; Peralta, J. E.; Ogliaro, F.; Bearpark, M.; Heyd, J. J.; Brothers, E.; Kudin, K. N.; Staroverov, V. N.; Kobayashi, R.; Normand, J.; Raghavachari, K.; Rendell, A.; Burant, J. C.; Iyengar, S. S.; Tomasi, J.; Cossi, M.; Rega, N.; Millam, J. M.; Klene, M.; Knox, J. E.; Cross, J. B.; Bakken, V.; Adamo, C.; Jaramillo, J.; Gomperts, R.; Stratmann, R. E.; Yazyev, O.; Austin, A. J.; Cammi, R.; Pomelli, C.; Ochterski, J. W.; Martin, R. L.; Morokuma, K.; Zakrzewski, V. G.; Voth, G. A.; Salvador, P.; Dannenberg, J. J.; Dapprich, S.; Daniels, A. D.; Farkas, Ö.; Foresman, J. B.; Ortiz, J. V.; Cioslowski, J.; Fox, D. J. *Gaussian 09, Revision D.01*, 2009.
[14] Buchholz, H. K.; Hylton, R. K.; Brandenburg, J. G.; Seidel-Morgenstern, A.; Lorenz, H.; Stein, M.; Price, S. L., Thermochemistry of Racemic and Enantiopure Organic Crystals for Predicting Enantiomer Separation. *Crys. Growth Des.* **2017,** *17*, 4676-4686.
[15] McDonagh, J. L.; Palmer, D. S.; van Mourik, T.; Mitchell, J. B. O., Are the Sublimation Thermodynamics of Organic Molecules Predictable? *J. Chem. Inf. Model.* **2016,** *56*, 2162-2179.
[16] Gavezzotti, A., *Theoretical Aspects and Computer Modeling of the Molecular Solid State*. John Wiley: Chichester, 1997.
[17] Palmer, D. S.; Mitchell, J. B. O. Is Experimental Data Quality the Limiting Factor in Predicting the Aqueous Solubility of Druglike Molecules? *Mol. Pharmaceutics* **2014**, *11*, 2962-2972.
[18] Palmer, D.S.; McDonagh, J.L.; Mitchell, J.B.O.; van Mourik, T.; Fedorov, M.V. First-principles calculation of the intrinsic aqueous solubility of crystalline druglike molecules. *J. Chem. Theory Comput.*, **2012**, *8*, 3322-3337.
[19] Price, S. L.; Leslie, M.; Welch, G. W. A.; Habgood, M.; Price, L. S.; Karamertzanis, P. G.; Day, G. M., Modelling Organic Crystal Structures using Distributed Multipole and Polarizability-Based Model Intermolecular Potentials. *Phys. Chem. Chem. Phys.* **2010,** *12*, 8478-8490.
[20] Tkatchenko, A.; Scheffler, M., Accurate Molecular Van Der Waals Interactions from Ground-State Electron Density and Free-Atom Reference Data. *Phys. Rev. Lett.* **2009,** *102*, 073005.
[21] Monkhorst, H. J.; Pack, J. D., Special points for Brillouin-zone integrations. *Phys. Rev. B* **1976,** *13*, 5188-5192.
[22] Nyman, J.; Yu, L.; Reutzel-Edens, S. M., Accuracy and reproducibility in crystal structure prediction: the curious case of ROY. *CrystEngComm* **2019,** *21*, 2080-2088.

[23] Reilly, A. M.; Cooper, R. I.; Adjiman, C. S.; Bhattacharya, S.; Boese, A. D.; Brandenburg, J. G.; Bygrave, P. J.; Bylsma, R.; Campbell, J. E.; Car, R.; Case, D. H.; Chadha, R.; Cole, J. C.; Cosburn, K.; Cuppen, H. M.; Curtis, F.; Day, G. M.; DiStasio Jr, R. A.; Dzyabchenko, A.; van Eijck, B. P.; Elking, D. M.; van den Ende, J. A.; Facelli, J. C.; Ferraro, M. B.; Fusti-Molnar, L.; Gatsiou, C.-A.; Gee, T. S.; de Gelder, R.; Ghiringhelli, L. M.; Goto, H.; Grimme, S.; Guo, R.; Hofmann, D. W. M.; Hoja, J.; Hylton, R. K.; Iuzzolino, L.; Jankiewicz, W.; de Jong, D. T.; Kendrick, J.; de Klerk, N. J. J.; Ko, H.-Y.; Kuleshova, L. N.; Li, X.; Lohani, S.; Leusen, F. J. J.; Lund, A. M.; Lv, J.; Ma, Y.; Marom, N.; Masunov, A. E.; McCabe, P.; McMahon, D. P.; Meekes, H.; Metz, M. P.; Misquitta, A. J.; Mohamed, S.; Monserrat, B.; Needs, R. J.; Neumann, M. A.; Nyman, J.; Obata, S.; Oberhofer, H.; Oganov, A. R.; Orendt, A. M.; Pagola, G. I.; Pantelides, C. C.; Pickard, C. J.; Podeszwa, R.; Price, L. S.; Price, S. L.; Pulido, A.; Read, M. G.; Reuter, K.; Schneider, E.; Schober, C.; Shields, G. P.; Singh, P.; Sugden, I. J.; Szalewicz, K.; Taylor, C. R.; Tkatchenko, A.; Tuckerman, M. E.; Vacarro, F.; Vasileiadis, M.; Vazquez-Mayagoitia, A.; Vogt, L.; Wang, Y.; Watson, R. E.; de Wijs, G. A.; Yang, J.; Zhu, Q.; Groom, C. R., Report on the sixth blind test of organic crystal structure prediction methods. *Acta Cryst. B 2016*, **72**, 439-459.
[24] Geatches, D.; Rosbottom, I.; Marchese Robinson, R. L.; Byrne, P.; Hasnip, P.; Probert, M. I. J.; Jochym, D.; Maloney, A.; Roberts, K. J., Off-the-shelf DFT-DISPersion methods: Are they now "on-trend" for organic molecular crystals? *J. Chem. Phys.* **2019,** *151*, 044106.
[25] Reilly, A. M.; Tkatchenko, A., Understanding the role of vibrations, exact exchange, and many-body van der Waals interactions in the cohesive properties of molecular crystals. *J. Chem. Phys.* **2013,** *139*, 024705-024705.
[26] Dolgonos, G. A.; Hoja, J.; Boese, A. D., Revised values for the X23 benchmark set of molecular crystals. *Phys. Chem. Chem. Phys.* **2019,** *21*, 24333-24344.
[27] Ambrosetti, A.; Reilly, A. M.; DiStasio, R. A.; Tkatchenko, A., Long-range correlation energy calculated from coupled atomic response functions. *J. Chem. Phys.* **2014,** *140*, 18A508.
[28] Stone, A. J., GDMA: A Program for Performing Distributed Multipole Analysis of Wave Functions Calculated Using the Gaussian Program System. *GDMA2.2* **2010**.
[29] Marchese Robinson, R. L.; Geatches, D.; Morris, C.; Mackenzie, R.; Maloney, A. G. P.; Roberts, K. J.; Moldovan, A.; Chow, E.; Pencheva, K.; Vatvani, D. R. M., Evaluation of Force-Field Calculations of Lattice Energies on a Large Public Dataset, Assessment of Pharmaceutical Relevance, and Comparison to Density Functional Theory. *J. Chem. Inf. Model.* **2019,** *59*, 4778-4792
[30] Marenich, A. V.; Cramer, C. J.; Truhlar, D. G. Universal Solvation Model Based on Solute Electron Density and on a Continuum Model of the Solvent Defined by the Bulk Dielectric Constant and Atomic Surface Tensions, *J. Phys. Chem. B*. **2009**, *113*, 6378-6396.
[31] Ho, J.; Klamt, H.; Coote, M. L. Comment on the Correct Use of Continuum Solvent Models *J. Phys. Chem. A* **2010**, *114*, 13442
[32] Ribeiro, R.F.; Marenich, A. V.; Cramer, C. J.; Truhlar, D. G. Use of Solution-Phase Vibrational Frequencies in Continuum Models for the Free Energy of Solvation *J. Phys. Chem. B,* **2011**, *115*, 14556–14562
